# Transposable Elements Are Major Contributors to the Origin, Diversification, and Regulation of Vertebrate Long Noncoding RNAs

**DOI:** 10.1371/journal.pgen.1003470

**Published:** 2013-04-25

**Authors:** Aurélie Kapusta, Zev Kronenberg, Vincent J. Lynch, Xiaoyu Zhuo, LeeAnn Ramsay, Guillaume Bourque, Mark Yandell, Cédric Feschotte

**Affiliations:** 1 Department of Human Genetics, University of Utah School of Medicine, Salt Lake City, Utah, United States of America; 2 Department of Human Genetics, University of Chicago, Chicago, Illinois, United States of America; 3 McGill University and Genome Quebec Innovation Center, Montréal, Canada; Harvard University, United States of America

## Abstract

Advances in vertebrate genomics have uncovered thousands of loci encoding long noncoding RNAs (lncRNAs). While progress has been made in elucidating the regulatory functions of lncRNAs, little is known about their origins and evolution. Here we explore the contribution of transposable elements (TEs) to the makeup and regulation of lncRNAs in human, mouse, and zebrafish. Surprisingly, TEs occur in more than two thirds of mature lncRNA transcripts and account for a substantial portion of total lncRNA sequence (∼30% in human), whereas they seldom occur in protein-coding transcripts. While TEs contribute less to lncRNA exons than expected, several TE families are strongly enriched in lncRNAs. There is also substantial interspecific variation in the coverage and types of TEs embedded in lncRNAs, partially reflecting differences in the TE landscapes of the genomes surveyed. In human, TE sequences in lncRNAs evolve under greater evolutionary constraint than their non–TE sequences, than their intronic TEs, or than random DNA. Consistent with functional constraint, we found that TEs contribute signals essential for the biogenesis of many lncRNAs, including ∼30,000 unique sites for transcription initiation, splicing, or polyadenylation in human. In addition, we identified ∼35,000 TEs marked as open chromatin located within 10 kb upstream of lncRNA genes. The density of these marks in one cell type correlate with elevated expression of the downstream lncRNA in the same cell type, suggesting that these TEs contribute to *cis*-regulation. These global trends are recapitulated in several lncRNAs with established functions. Finally a subset of TEs embedded in lncRNAs are subject to RNA editing and predicted to form secondary structures likely important for function. In conclusion, TEs are nearly ubiquitous in lncRNAs and have played an important role in the lineage-specific diversification of vertebrate lncRNA repertoires.

## Author Summary

An unexpected layer of complexity in the genomes of humans and other vertebrates lies in the abundance of genes that do not appear to encode proteins but produce a variety of non-coding RNAs. In particular, the human genome is currently predicted to contain 5,000–10,000 independent gene units generating long (>200 nucleotides) noncoding RNAs (lncRNAs). While there is growing evidence that a large fraction of these lncRNAs have cellular functions, notably to regulate protein-coding gene expression, almost nothing is known on the processes underlying the evolutionary origins and diversification of lncRNA genes. Here we show that transposable elements, through their capacity to move and spread in genomes in a lineage-specific fashion, as well as their ability to introduce regulatory sequences upon chromosomal insertion, represent a major force shaping the lncRNA repertoire of humans, mice, and zebrafish. Not only do TEs make up a substantial fraction of mature lncRNA transcripts, they are also enriched in the vicinity of lncRNA genes, where they frequently contribute to their transcriptional regulation. Through specific examples we provide evidence that some TE sequences embedded in lncRNAs are critical for the biogenesis of lncRNAs and likely important for their function.

## Introduction

There is a growing appreciation that the functional repertoire of metazoan genomes includes much more than protein-coding sequences [1]–[3]. Recent functional genomic studies have revealed, in particular, the widespread occurrence, bewildering diversity, and functional significance of noncoding RNA [4]. In addition to small regulatory RNAs, such as tRNAs or microRNAs, the genome encodes a myriad of long noncoding RNAs (lncRNAs) that are greater than 200 nt in length [for review: 5]–[7]. The most recent, though still conservative, catalogues predict between 5,000 and 10,000 discrete lncRNA loci in the human genome [8]–[10]. The majority of lncRNAs in these manually curated reference sets are intergenic units often referred to as large intergenic noncoding RNAs (lincRNAs) because they do not overlap with known protein-coding genes. Comparable numbers of lncRNA loci are expected to occur in the mouse and other vertebrate genomes [9], [11]–[16] and hundreds of loci with similar properties have also been identified in model invertebrates such as *Drosophila melanogaster* [17] and *Caenorhabidtis elegans* [18], as well as in the model plant *Arabidopsis thaliana* [19].

Although once dismissed as transcriptional ‘noise’, there is mounting evidence that many lncRNAs are important functional molecules engaged in diverse regulatory activities. First, the majority of functionally characterized lncRNAs exhibit precise spatiotemporal patterns of expression and, often, discrete cellular localization [9], [11]–[13], [20]–[25]. Second, the structure, biogenesis and processing of lncRNAs are very similar to that of protein-coding genes and indicate that most lncRNAs are produced from independent transcription units. For example, lncRNAs are typically transcribed by RNA polymerase II, under the control of diverse combinations of transcription factors that actively bind to promoters and enhancers, with canonical chromatin modifications [10]–[12], [26], [27]. LncRNA transcripts are also alternatively spliced, polyadenylated, and subject to other post-transcriptional modifications [10], [12], [28]. Third, lncRNA exons generally display a clear signal of purifying selection, implying structural and/or functional sequence constraint, albeit less stringent than on protein-coding exons [10], [12], [29]–[33]. Moreover, some lncRNA genes are evolutionarily ancient. A small but increasing number of loci orthologous to human lncRNAs have been identified in the mouse, and the origins of some human lncRNAs can be traced to the common ancestor of mammals, amniotes, or even vertebrates [9], [10], [14], [16], [34], [35]. Finally, a growing body of genetic and biochemical work on individual lncRNAs, as well as more systematic approaches to explore lncRNA function and their association with disease, point to crucial regulatory activities, notably in cell differentiation and embryonic development [for review: 7], [23], [36]–[44].

While the precise molecular functions of lncRNAs are still poorly understood, even less is known about their origin and evolution. Four non-mutually exclusive hypotheses have been proposed for the emergence of lncRNAs [6], [14]: (i) transformation of a protein-coding genes; (ii) duplication of another lncRNA; (iii) de novo origin from sequences previously untranscribed or devoid of exonic sequences; (iv) emergence from transposable element (TE) sequences. Individual examples illustrating each of these mechanisms have been described. For example, *Xist*, a lncRNA controlling mammalian X inactivation, originated in the eutherian ancestor from a mixture of exons derived from a decayed protein-coding gene [45] together with a variety of transposable elements (TEs) progressively accumulated and ‘exonized’ at this locus [46]. However, with the exception of a few emblematic and intensively studied lncRNAs such as *Xist*, the origins of most lncRNAs remain elusive. In one of the most systematic efforts to trace the origins of lncRNAs, Ulitsky et al. [14] found that a minority (∼15%) of zebrafish lncRNAs showed significant sequence similarity to another lncRNAs or protein-coding genes in the zebrafish genome. Likewise, Derrien et al. [10] reported that human lncRNAs rarely have extensive sequence similarity to each other outside of shared repetitive elements. Collectively these observations suggest that, in contrast to protein-coding genes, novel lncRNA genes do not commonly arise by duplication, but rather may emerge *de novo* from previously non-exonic sequences and/or from TEs.

TEs occupy a substantial fraction of vertebrate genomes (e.g. at least half of the human genome [47], [48]) and are increasingly recognized as important players in the origin of functional novelties [for review: 49]–[52]. Several instances of TEs co-opted for cellular function on a genome-wide scale have been documented, notably as a source of *cis*-elements regulating adjacent host genes, such as promoters [53], [54], transcription factor binding sites [55]–[57], enhancers [58], [59] or insulators [60], [61]. TEs can also be ‘exonized’ into novel coding and non-coding exons [for review: 49], [62], [63]. As a source of non-coding exons, TEs have been shown to contribute substantially to untranslated regions [64]–[67] and to alternatively spliced exons of protein-coding genes [66]–[70], as well as to microRNA genes [71], [72]. In this study we provide evidence for the widespread involvement of TEs in the assembly, diversification, regulation, and potential function of lncRNAs.

## Results

### Datasets

We focus on three vertebrate species -human, mouse and zebrafish- for which extensive lncRNA datasets are available (Table 1). Each set has been ‘manually’ curated based on a combination of bioinformatics and high-throughput genomics experiments, such as deep sequencing of polyadenylated RNAs (RNA-seq), chromatin state maps and cap-analysis of gene expression (CAGE) or paired-end ditags to determine transcript termini. For human, we primarily analyzed the most recent Gencode catalog of lncRNAs (v13) produced from 15 cell lines as part of the ENCODE project [10], [73], [74]. We replicated most analyses on another large set of lncRNAs assembled by Cabili et al. [9] from 24 human tissues and cell types. Importantly, the Gencode and “Cabili” sets differ slightly in the way they were curated and they are only partially overlapping [10]. Indeed we found that 64.9% of the Gencode v13 genes have no overlap with genes in the Cabili set, and conversely 47.3% of the Cabili genes have no overlap with the Gencode v13 set. While the Cabili set only contains “intergenic” (l*i*ncRNA) units (no overlap with known protein-coding genes), the Gencode catalog includes also “genic” lncRNAs, i.e. those overlapping or nested within protein-coding genes [10], Figure S1. Thus, these two sets may be viewed as complementary rather than redundant, acting as “biological replicates” for our study. For mouse, we primarily studied lincRNAs from Ensembl (release 70) and replicated some analyses on lincRNAs from adult liver tissue compiled by Kutter et al [16]. For zebrafish, we merged the sets of developmentally expressed lncRNAs from Pauli et al. [24] and lincRNAs from Ulitsky et al. [14] (see Methods for more details).

**Table 1:** Number of genes and transcripts in studied datasets.

Datasets	Genes#	Transcripts
Human, Gencode v7 [10]	9,277	14,880
Human, Gencode v13	12,393	19,835
Human, lincRNAs, Cabili et al. (2011) [9]	8,263	14,353
Mouse, lincRNAs, Ensembl release 70	1,671	2,167
Mouse, lincRNAs, Kutter et al. (2012) [16]	293	293
Zebrafish [14], [24]	1,402	1,780

# For zebrafish gene annotation, see Methods. Other numbers are from the datasets themselves.

### A substantial fraction of vertebrate lncRNAs contain exonized TE sequences

**Figure 1:**
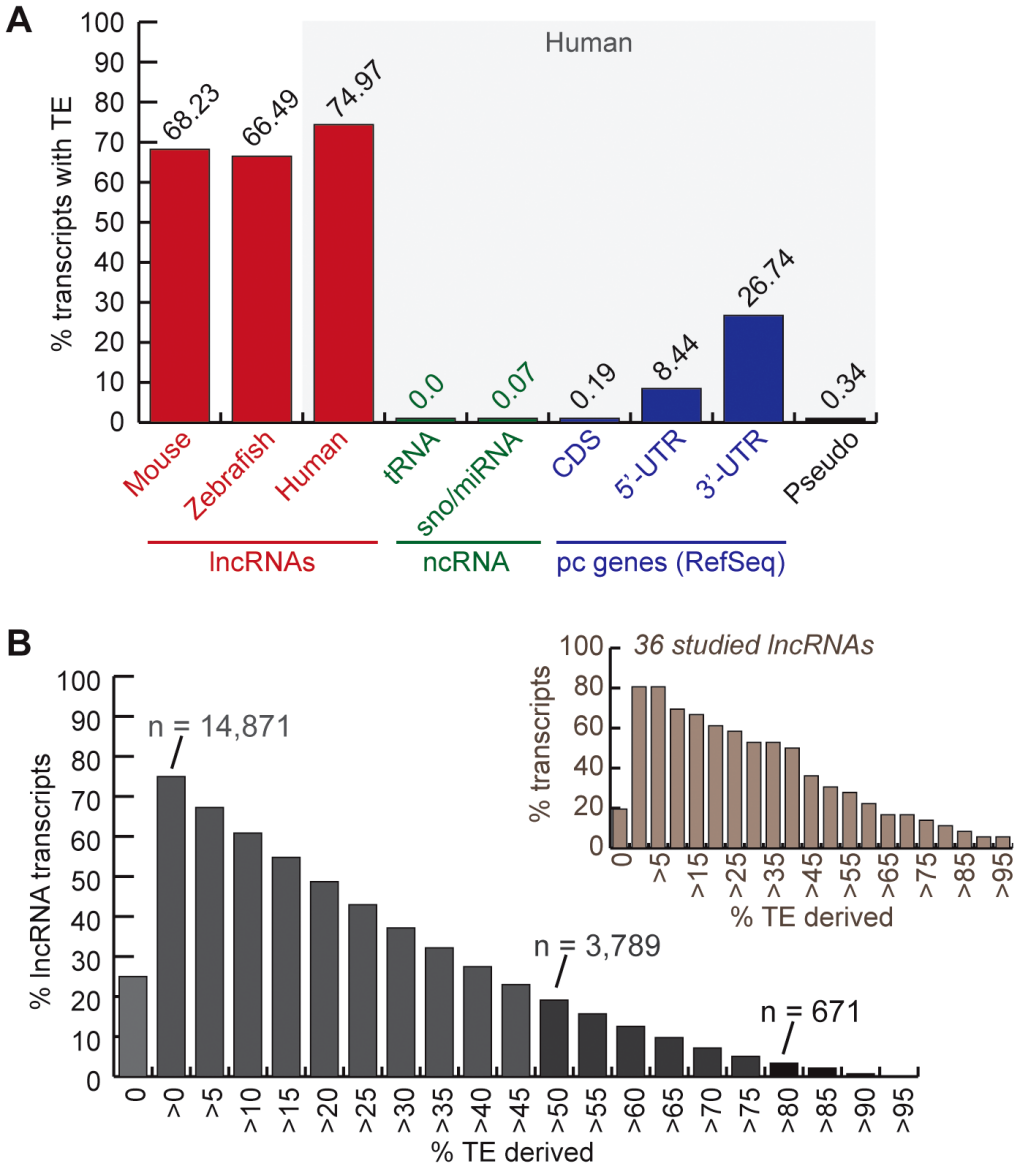
TE occurrence in lncRNAs. See text, Methods and Table 1 for more details about lncRNA datasets. A. Percentage of transcripts with at least one exon overlapping with a TEs fragment (at least 10 bp). In red, lncRNAs (human = Gencode v13; mouse = both sets). Rest corresponds to human Refseq 57: in green, small non-coding RNAs (tRNAs and sno/miRNAs); in blue, protein-coding genes (pc genes) separated in exon types (coding and non-coding = UTRs); in black, pseudo = pseudogenes. B. Distribution of percentage of human lncRNA transcripts (Gencode v13) derived from TEs (more than 0% to more than 95%). The number of transcripts with more than 80% and more than 50% TE-derived DNA exons are indicated. Distribution is also shown for the subset of 36 studied lncRNAs presented in Table 2 and Table S2.

We inferred the TE content of lncRNAs by calculating the fraction of lncRNA transcripts with exons overlapping at least 10-bp of DNA annotated as TE by RepeatMasker (see Methods). We found that 75% of human (Gencode v13) lncRNA transcripts contain an exon of at least partial TE origin, which is considerably much higher than any other type of RNAs such as small ncRNAs (tRNAs, sno/miRNAs), pseudogenes, coding exons (less than 1%), as well as UTRs, the non-coding parts of mRNAs (Figure 1A). The median length of TE-derived fragments in human lncRNAs is 112 nucleotides and the average is 150 nucleotides. While the majority of human lncRNA transcripts are comprised of a relatively small percentage of TE-derived sequences, 3,789 out of 19,835 transcripts examined (∼19%) are composed of ≥50% of TE-derived sequences (Figure 1B). Similarly, 68.23% and 66.5% of mouse and zebrafish lncRNA transcripts, respectively, contain exonic sequences of at least partial TE origin (Figure 1A).

**Figure 2:**
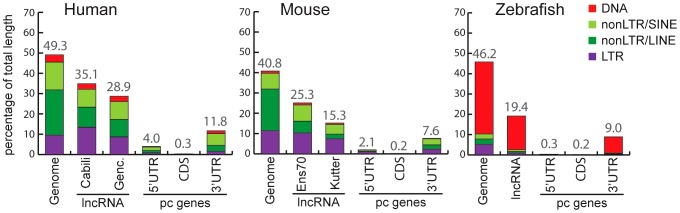
Coverage of different TE classes in genome, lncRNA, and protein-coding exons in human, mouse, and zebrafish. For genomes, total length (100%) corresponds to total length of assembly without gaps (human: 2,897 Mb. Mouse: 2,620 Mb. Zebrafish: 1,401 Mb). For lncRNAs, total length of genomic projection of all of exons are considered (human, Genc. = Gencode v13: 14.2 Mb. Human, Cabili set: 8.5 Mb. Mouse, Ens70 = Ensembl 70: 2.8 Mb. Mouse, Kutter: 0.15 Mb. Zebrafish: 2.3 Mb). For protein coding genes (pc genes), total length of CDS exons, 5′ and 3′UTR respectively are as follow: human, 30.9 Mb, 5.2 Mb, 24.6 Mb. Mouse: 30.5 Mb, 4.0 Mb, 21.6 Mb. Zebrafish: 19.1 Mb, 33.6 Mb, 12.5 Mb. Only pc genes from Refseq annotations with CDS and UTR features are considered (see Methods). Percentage of coverage of all TEs is indicated above bars.

To measure the total coverage of TE-derived sequences in lncRNA exons in each species, we intersected TE annotations from RepeatMasker (with a minimum overlap of 10 bp, see Methods) with the genomic coordinates of all lncRNA exons, and for comparison, with UTRs and coding exons of RefSeq protein-coding genes. The results show that, in all three species TE coverage is considerably higher for lncRNA exons than for protein-coding exons, but still lower than in the whole genome (Figure 2). The fraction of lncRNA exon sequence covered by TEs is also at least twice higher than in their UTRs.

We noticed that the Cabili set [9], which consists exclusively of intergenic units (lincRNAs) shows greater TE coverage (35.1%; Figure 2) than the Gencode v13 set (28.9%; Figure 2), suggesting that intergenic lncRNAs may have a higher TE content than ‘genic’ lncRNAs (i.e. those overlapping protein-coding genes). Consistent with this idea, the TE coverage of intergenic lncRNAs in the Gencode v13 set is 31.8%, while genic lncRNAs are comprised of 25.9% of TE-derived sequences (Table S1). Thus, human lincRNA transcripts tend to be richer in TE sequences than genic lncRNAs. We wondered whether this trend could merely reflect a higher TE density in intergenic regions in general. It does not appear to be the case because the TE coverage of introns and surrounding sequences of Gencode intergenic lncRNAs is similar to that of protein-coding genes or genic lncRNAs (Table S1). These observations suggest that TEs are more prevalent in intergenic lncRNAs than in genic lncRNAs.

**Figure 3:**
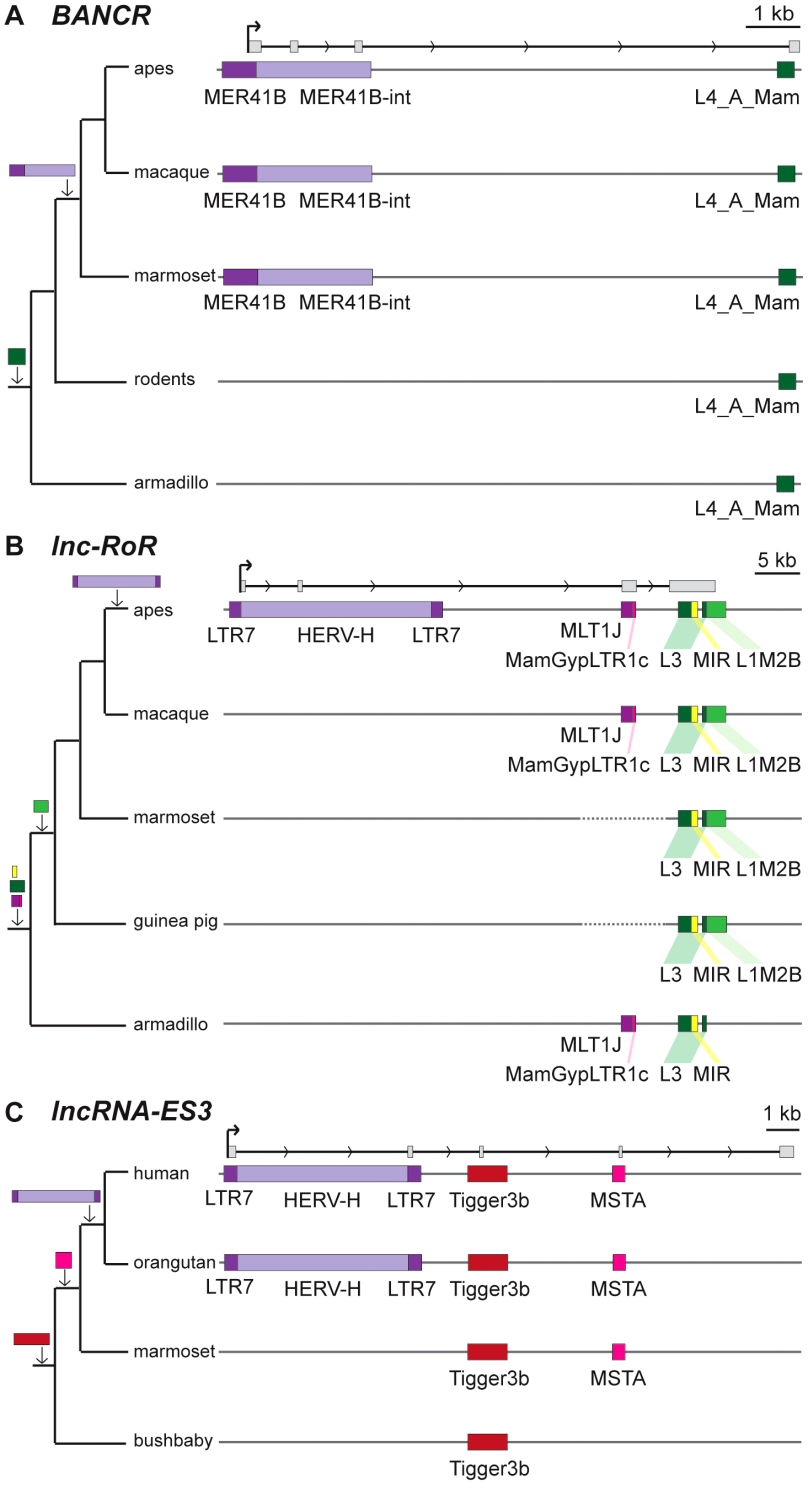
Examples of lncRNAs with embedded TEs. Genomic DNA is represented as a grey line, transcripts are represented by a black line, with arrows showing sense of transcription and in grey boxes the exons of the mature transcript. TEs as colored boxes (orange-red: DNA TEs. Yellow: SINEs. Pink-purple: LTR/ERVs. Green: LINEs). Only TEs overlapping with lncRNA exons are represented. See also Table S2 for details of TEs in these lncRNAs. A. *BANCR* [79]. B. *lnc-RoR* [41]. Apes = gibbon, gorilla, orangutan, bonobo, chimpanzee, human. C. *lnc-ES3* [43].

A survey of individual human lncRNAs previously characterized in the literature recapitulates the omnipresence and high prevalence of TE sequences we detect in the *ab initio* lncRNA catalogs (Figure 1B, Table 2 and for an expanded version, Table S2). The presence of exonized TEs has been reported for some of these lncRNAs, such as *XIST* [46], *UCA1* [75], *HULC* [76], *PCAT-14* [77] and *SLC7A2-IT1A/B* [78]. But for the majority, there has been no previous mention of embedded TEs, even though some of these mature lncRNA transcripts are almost entirely composed of TE sequences. For example, the first three exons (out of four, i.e. ∼86% of the sequence) of the mature transcript of *BANCR*, which is involved in melanoma cell migration [79], are derived from a MER41 long terminal repeat/endogenous retrovirus (LTR/ERV) element (Figure 3A). The mature transcript of *lincRNA-RoR*, which has been shown to modulate the reprogramming of human induced pluripotent stem cells [41], is made from an assemblage of 6 different TEs together accounting for 2,057 nt (79.7%) of its length (Figure 3B) [see also ref. 7]. Importantly, the structures of *BANCR* and *lincRNA-RoR* transcripts have been validated by a combination of RACE and RT-PCR experiments and their function investigated by siRNA knockdowns and rescue experiments [41], [79]. These transcripts were independently retrieved and their structure accurately predicted in the Cabili and Gencode v13 sets, respectively. In mouse, *Fendrr* lincRNA, which has a very restricted pattern of expression in lateral mesoderm [80], initiates within a MTEa (ERVL-MaLR) and 4 different TEs account for 808 nt (33.7%) of its length (data not shown). In summary, our analyses point to an extensive contribution of TEs to the content of mature lncRNA transcripts, including many of those with established regulatory functions.

**Table 2:** TE content of known lncRNAs in human.

Gene/ID#	Range of % of TE based DNA of mature transcripts when applies	TSS in TE: class (number of transcripts)	polyA in TE: class (number of transcripts)	number of transcripts: with TE/total
*PCAT14*	99.6–99.96	LTR (2)	LTR (2)	2/2
*BANCR*	86.4	LTR (1)	-	1/1
*Lnc-RoR*	79.7	LTR (1)	LINE (1)	1/1
*PTCSC3*	60.6	-	LTR (1)	1/1
*BACE1-AS*	56.4	SINE (1)	-	1/1
*UCA1*	51.8	LTR (1)	-	1/1
*HULC*	45.7	LTR (1)	-	1/1
*LINC00458 (LncRNA-ES3)*	45.3–57	LTR (3)	-	3/3
*ncRNA-7 (LINC00651)*	41.1			
*lincRNA-p21*	40.9	-	SINE (1)	1/1
*NEAT1*	38.1	-	-	1/2
*PCAT1*	37.1–81.7	-	DNA (1)	2/2
*AK023948*	29.7	-	-	1/1
*KCNQ1OT1*	29.5	-	-	1/1
*Tie-1AS*	24.4–96.3	LINE (2)	-	3/3
*PTENP1*	21	-	LINE (1)	1/1
*linc-CCDC90A-1 (LncRNA-ES1)*	13.8	-	LTR (1)	1/1
*OIP5-AS1 (Cyrano)*	12.5–64.7	-	LTR (1); SINE (3); LINE (1)	10/10
*SLC7A2-IT1*	11.6–18.6	-	LINE (2)	2/2
*HOTAIRM1*	9	-	-	1/5
*BIRC6 (megamind)*	8.9	LINE (1)	-	1/1
*HAR1*	7.2	-	DNA (1)	1/1
*BDNFOS (BDNF-AS)*	6.4–71.1	-	-	9/11
*MEG3*	5.6–47.1	SINE (2); LINE (1)	SINE (6)	23/28
*GAS5*	5.3–40.9	-	SINE (3)	23/29
*Xist*	5.3–17.4	-	-	2/8
*MALAT1/NEAT2*	4.1–7.7	-	-	2/3
*ANRIL (CDKN2B-AS1, p15AS1, Mycn)*	4–41.1	SINE (1)	SINE (7); DNA (1)	16/17
*TUG1*	1.9–40.6	-	LTR (1)	6/7

# Known lncRNAs with no detectable exonized TE are not shown, but include: *HOTAIR*, *Zeb2AS1*, *TERC*, *PANDA*, *H19*, *LncRNA-ES2* and *UNCA-RC*. See Table S2 for an expanded version of this table and references.

### TE sequences in lncRNAs evolve under modest yet greater functional constraint than their non–TE sequences

**Figure 4:**
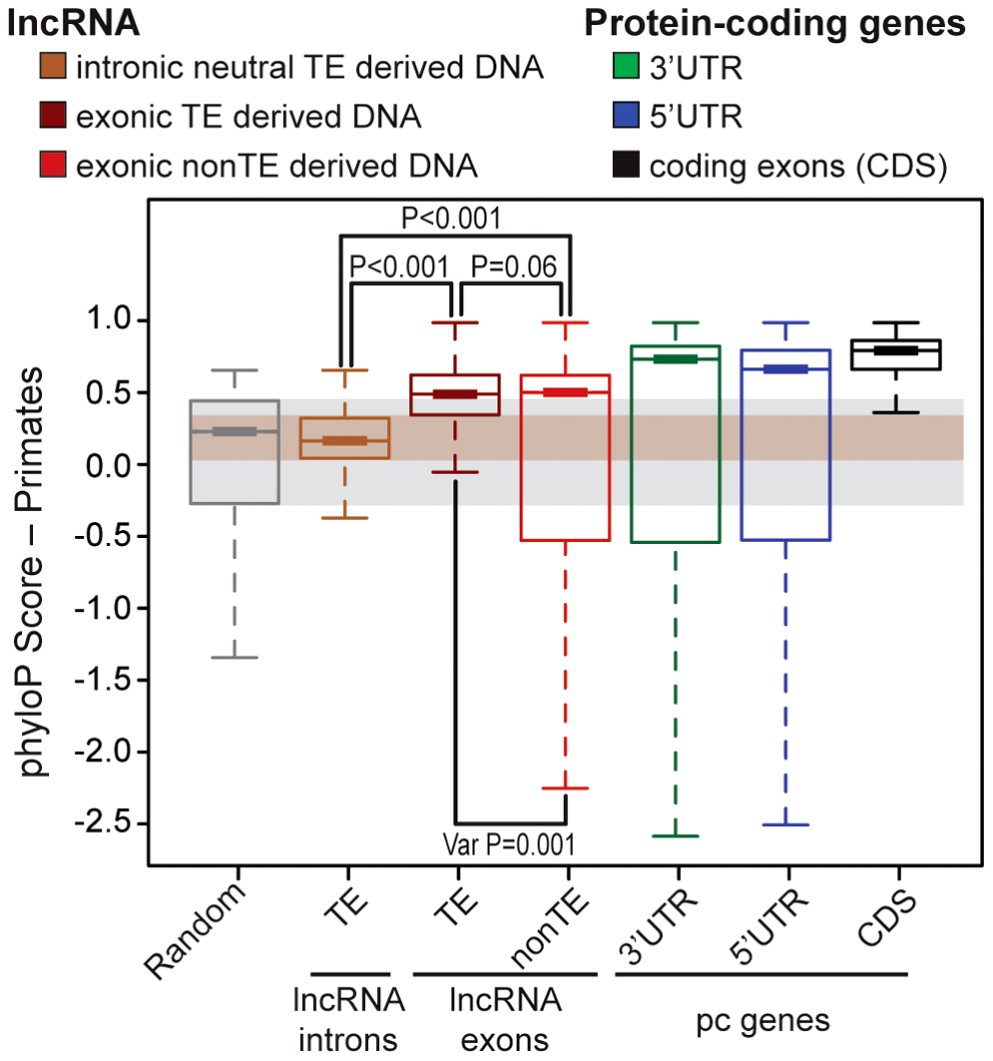
Evidence of purifying selection in TE–derived DNA transcribed as lncRNAs. LncRNAs correspond to Gencode v13 (human) and protein coding genes to Refseq 57 (human, 20,848 genes). Boxplots show primate PhyloP scores computed in order to compare the conservation of different sets (see upper panel). Random set is size and number-matched for TE-derived DNA in lncRNA exons. Intronic lncRNA TEs correspond to TE-derived DNA in lncRNA introns that don't overlapp with splicing sites and all annotated chromatin marks were removed (see Methods), in order to obtain a most neutral set [inactive chromatin, see 32]. Statistical test used: permutation test with 1000 permutations were performed in R. Boxplots depicts the median upper (75%) and lower (25%) quantiles. The whiskers extend beyond the upper and lower quantile by 1.5× the inner quartile range. Outliers have been removed for visualization.

We next sought to evaluate the functional potential of TEs embedded in lncRNA transcripts. Several studies have reported that lncRNA exons show a signature of evolutionary constraint based on interspecies conservation [10], [12], [29], [30] as well as reduced nucleotide diversity in the human population [31]–[33] compared to randomly sampled regions of the genome or surrounding non-exonic sequences. Nonetheless, the level of constraint acting on lncRNA exons assessed through these analyses was much weaker than on protein-coding exons, presumably reflecting greater malleability of lncRNAs. To compare the level of selective constraint acting on TE-derived sequences to non-TE derived sequences in human lncRNAs (Gencode v13) and to various other types of genomic regions, we aggregated conservation scores per nucleotide calculated by phyloP across an alignment of 10 primate genomes (see Methods). As expected, we found that both TE and non-TE sequences in lncRNA exons were much less conserved than coding exons or UTRs of protein-coding transcripts (Figure 4A). Strikingly though, we found that TE sequences within lncRNA exons were significantly more conserved than either a size-matched random set of genomic regions or a neutral set of TE sequences residing in lncRNA introns (*permutation test*, p<0.001) (Figure 4A). Interestingly, TE-derived sequences are also more conserved than non-TE sequences according to this analysis (*permutation test*, p<0.06) and have significantly less variance in phyloP scores with fewer fast evolving sites than non-TE sequences in lncRNAs (*permutation test*, p<0.001) consistent with greater functional or structural constraints acting on TE-derived sequences in lncRNA genes than non-TE derived sequences. Hence, there appears to be enough functional constraint acting on TE-derived regions of lncRNAs to yield a detectable signal of purifying selection when these sequences are taken as a whole and compared across primate species. These data are consistent with the idea that some of the TE sequences embedded in lncRNAs are evolving under functional and/or structural constraints.

### TEs functionally contribute to every step in lncRNA biogenesis

**Figure 5:**
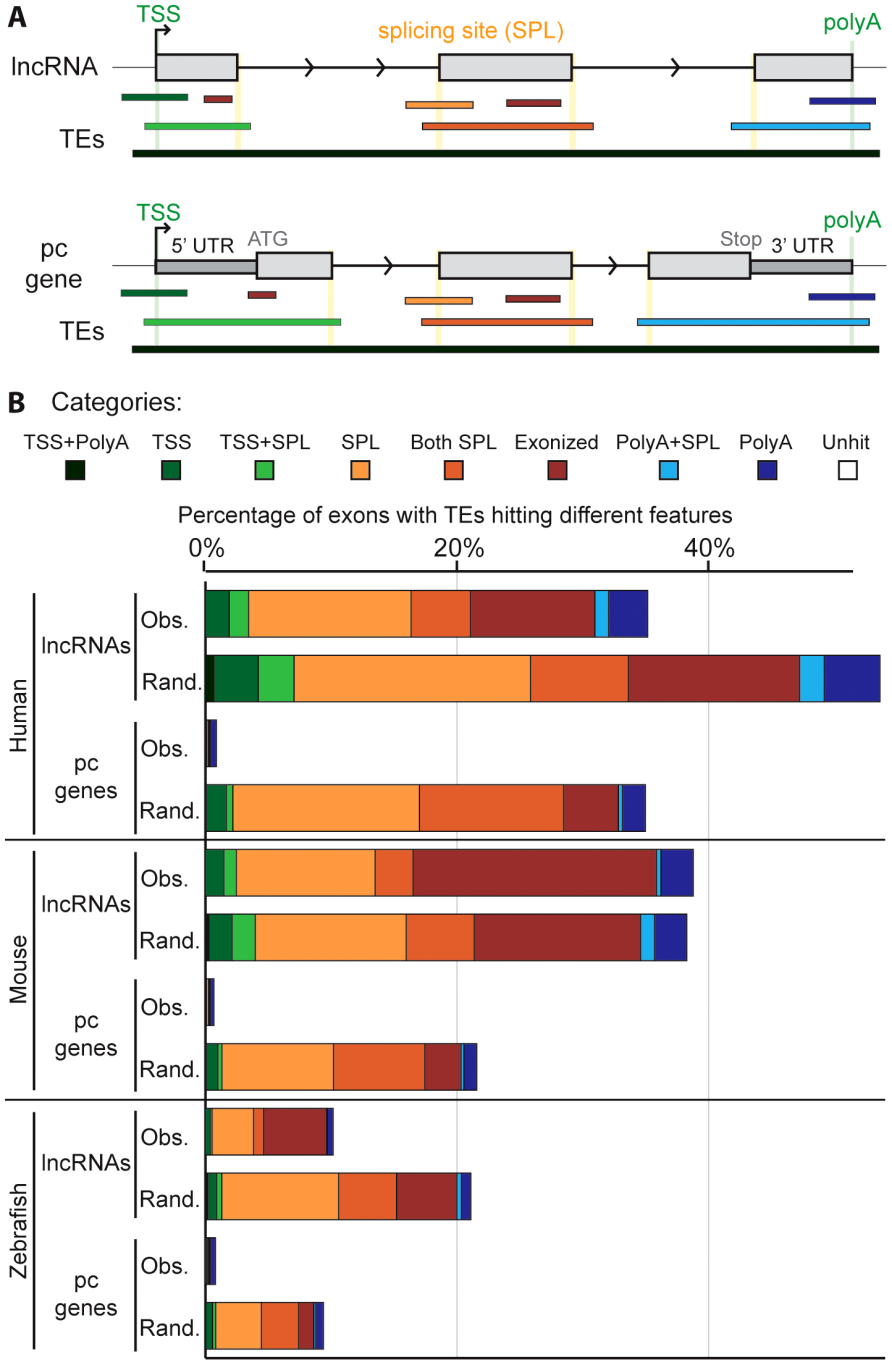
Contribution of TEs to different gene features of lncRNAs. A. Schematic of the type of overlap between TE and lncRNA sequences. Upper panel shows an idealized lncRNA transcription unit, and lower panel shows a protein-coding gene (only genes with annotated 5′ and 3′UTRs were analyzed; see Methods). Exons (grey boxes) overlapping a TE are categorized based on the type of overlap: the TE may provide functional feature(s), as a transcription start site (TSS), the first exon (including TSS and splicing site: TSS+SPL), a splicing site (SPL), a middle exon (including the 2 splicing sites (Both SPL), a polyadenylation site (polyA), the last exon (including splicing site and PolyA: PolyA+SPL). A TE not overlapping with any feature is called exonized. B. Comparison between observed (Obs) and random (Rand) distribution (see Methods). Note that a given exon can belong to several categories since a given TE can hit different exons and therefore be counted multiple times. Unhit exons correspond to exons with no TE overlap. Human: lncRNAs from Gencode v13. Mouse: lincRNAs from Ensembl release 70. With the exception of ‘exonized’, ‘TSS’ and ‘polyA’ categories in mouse (p-values = 1, 0.001 and 0.298 respectively) and ‘exonized’ category in zebrafish (p-value = 0.001), the p-values were systematically <0.0001.

To investigate the possible functional contributions of TEs to lncRNAs, we examined where TE segments and exons overlap in lncRNA genes. We defined eight categories of overlap (Figure 5A). For example, a TE may overlap with the internal part of an exon (called ‘exonized’ in Figure 5), a transcription start site (TSS), a polyadenylation (polyA) site, one or multiple splice sites, or a combination of these categories. We found that TE segments frequently overlap with and thereby directly contribute large quantities of these functional features to lncRNAs whereas they only rarely do so in protein-coding transcripts (Figure 5B, Table S3). For example, 22.5% and 29.9% of non-redundant TSS and polyA sites, respectively, used by lncRNA transcripts in the human Gencode v13 set are provided by TEs (18.2% and 19.0% in the Cabili set). By contrast, TEs contribute only 1.7% of TSS and 7.9% of polyA sites for full-length cDNAs of protein-coding genes. In total, we identified 29,519 and 19,214 TE-derived functional features (TSS, polyA and splice sites) in Gencode v13 and Cabili lncRNA sets respectively (Tables S7 and S8). For the Gencode set, this represents 9 times more TE-derived features than in protein-coding transcripts despite having 1.5 times more protein-coding transcripts available for analysis. We also retrieved high percentages of non-redundant TSS and polyA sites derived from TEs in the mouse Ensembl lincRNA set (18.5% and 24.7% respectively, see also Table S9), mouse “Kutter” set (12.3% and 16.7% respectively, see also Table S10), as well as in the zebrafish set (12.4% and 12.7% respectively, see also Table S11).

We next sought to assess whether the relative contribution of TEs to the different categories of genic features differ from a random model of overlap based on the frequency and coverage of TEs in the genome. In other words, we wondered to what extent the level and type of overlap might reflect the mere abundance of TEs in the genome. To investigate this question, we compared the percentage of exons containing different TE-derived features for lncRNAs and protein-coding transcripts to 5,000 simulations where we maintain exon positions but reshuffled randomly the coordinates of TE segments in each genome (see Methods). The results (Figure 5B and Table S3) reveal a similar pattern for all three species: with the exception of the ‘exonized’ and ‘polyA’ categories in mouse, the reshuffled sets yield significantly (p<0.001 or p<0.0001, see Figure 5B and Methods) greater overlap of TEs with every type of exonic features examined than with the actual TEs observed in the genome (compare “observed” and “random” profiles in Figure 5B). However, the gap between observed and randomized TE sets was much more pronounced for protein-coding transcripts than for lncRNAs (Figure 5B). These data suggest that the contribution of TEs to functional genic features is much greater for lncRNA than for protein-coding loci, but are still less than expected based on their sheer genomic abundance. We presume that this pattern reflects the action of natural selection to preserve lncRNA structure and function. The more pronounced gap between observed and random TE overlaps for protein-coding exons is consistent with the greater functional constraint (Figure 4) and stronger resistance to TE accumulation, in coding and UTR sequences than in lncRNAs (Figure 1 and Figure 2). Consistent with this idea, TEs inserted in lncRNA exons tend to be older than in the genome, even though here again this trend is not as strong as the one observed for protein-coding exons (Figure S5).

### Do all TEs contribute equally to lncRNAs?

**Figure 6:**
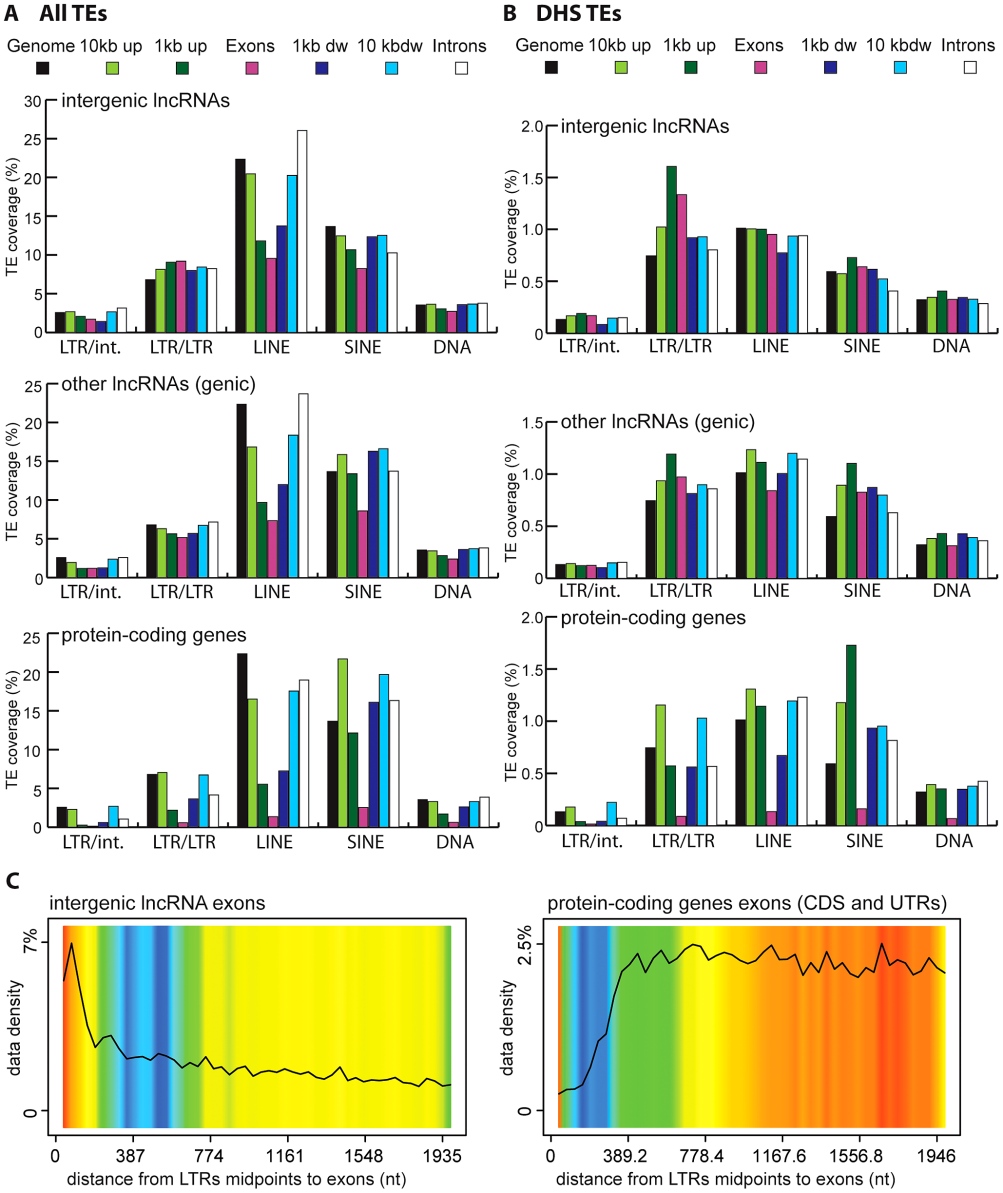
TE amounts and types in human lncRNA and their surrounding regions. Regions are genome, intergenic regions and exons. In the case of protein coding genes, exons include UTR exons as well as coding exons. 1 or 10 kb up and dw = intergenic regions up to 1 or 10 kb upstream of the TSS and downstream of the polyA respectively. Any annotated exons (RefSeq and Gencode v13 lncRNAs) have been subtracted from intergenic and intronic regions. A. Coverage of all TEs. LncRNA set corresponds to Gencode v13, separated in lincRNA transcripts (intergenic) and genic transcripts. Coverage is calculated as described for Figure 2 and in Methods and is shown per TE class (LTR/ERV, nonLTR/LINE, nonLTR/SINE, DNA) with an additional separation between ERVs (LTR/LTR) and internal parts (LTR/int) of ERV elements. B. Same as A, except that only TEs that overlap with DNaseI hypersensitive sites (‘TE-DHS’) are considered (see Methods). C. Heatmap of distance between LTR and lincRNA (left) and protein-coding genes (right) aggregated for all chromosomes (Jaccard test see Methods). The x-axis is the alignment of all reference features (protein coding exons and lncRNAs). The line depicts the total percentage of TEs found along the reference feature. The color quantifies the departure from null distribution generated from permutation. “Hot” (red) and “cool” (blue) colors mean that there was more or less TEs observed at a given position than by chance, respectively. All p–value <0.001.

**Figure 7:**
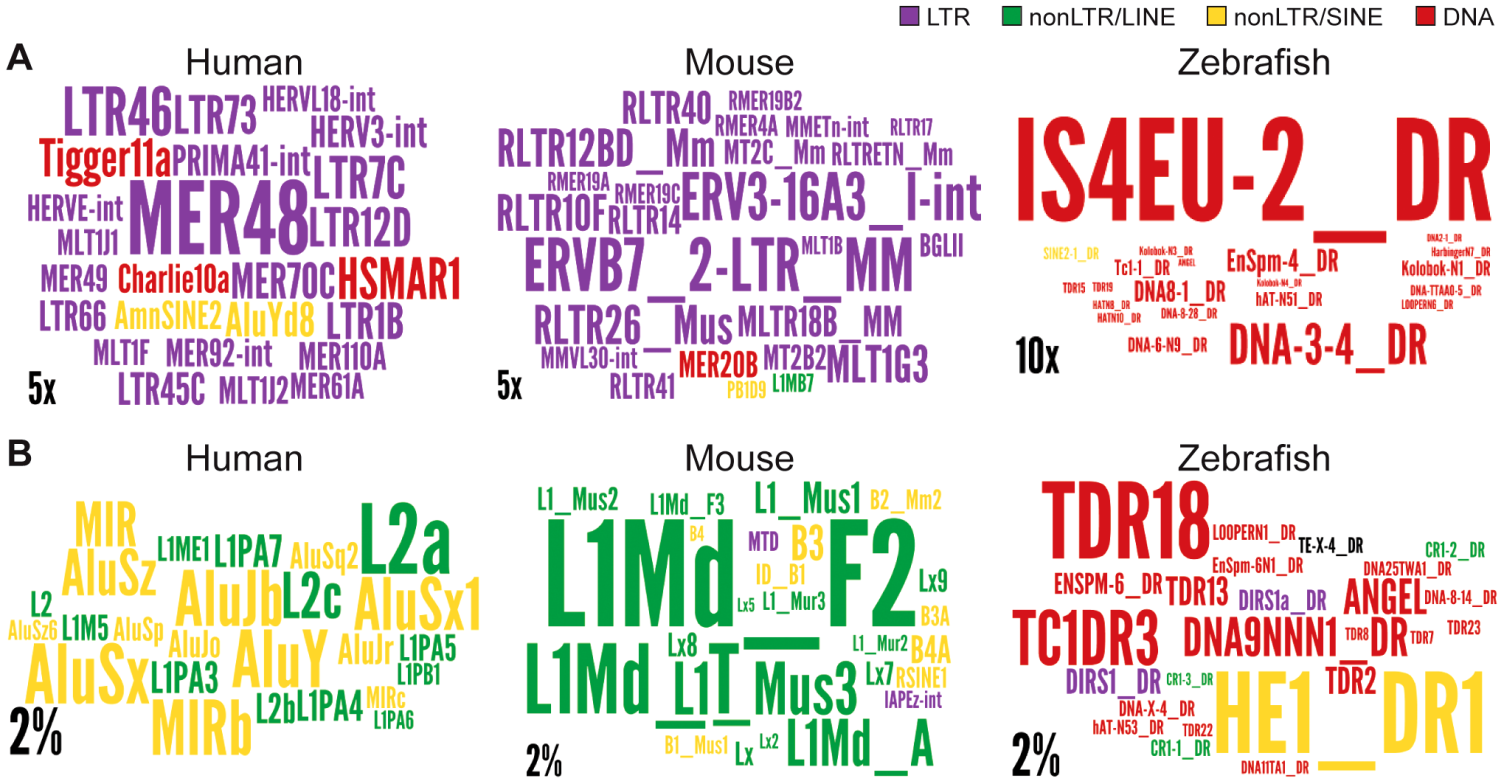
Wordle representation of the most enriched TE families in lncRNAs. Colors refer to different TE classes: purple = LTR, green = LINE, yellow = SINE, red = DNA. A. See also Figure S4. Human lncRNA set is from Gencode v13, mouse is from Esembl. The expected and observed counts of fragments corresponding to each TE are calculated using RepeatMasker output (see Methods). Observed values are obtained by considering overlapping TEs lncRNA exons. Expected values are calculated based on the overall density of each TE family in the genome according to the RepeatMasker output assuming a random distribution of TE family members throughout the genome.). Only families statistically enriched in term of counts (fragment numbers) are kept (at least p-value<0.05, binomial distribution test) and only ratios above 2 are represented on wordle. For human sets, TEs with less than 5 fragments in lncRNAs are removed, 4 fragments for mouse and zebrafish. Size of the TE family name is proportional to its over-representation (scales of 5× or 10× are represented). B. Visual representation of the 25 most abundant TE families in the 3 species. Size of the TE family name is proportional to its percentage of TE derived DNA in the genome (scale of 2% is represented).

Vertebrate TEs can be divided into four major types: short interspersed elements (SINEs), long interspersed elements (LINEs), LTR/ERV elements, and DNA transposons; and each of these subclasses comprises multiple families. Because each subclass and family of TEs has its own functional properties and evolutionary history, we were interested to see if they have made different contributions to lncRNAs. Overall we observed that all four major TE types contribute to lncRNA exons roughly proportionally to their representation in the genome (Figure 2). While the human and mouse genomes are largely dominated by SINEs and LINEs, the zebrafish genome is dominated by DNA transposons. These genomic TE landscapes are mirrored in the TE content of their lncRNA repertoires (Figure 2). The most striking departure from this general trend is apparent in human and mouse lncRNAs, where LINEs seem under-represented and LTR/ERV elements over-represented (Figure S1). Guided by these preliminary observations, we compared in more detail the content (nucleotide coverage and copy counts) of different TE types in exons, introns, and flanking regions of the 3 species lncRNAs and protein-coding genes (Figure 6B and Figure S2). Consistent with the action of purifying selection to purge TE insertions within or in close proximity to genes, we observe a markedly decreased coverage of TEs in exons and proximate genic regions (1 kb upstream or downstream) compared to introns and more distal regions (1–10 kb upstream or downstream) or to their total coverage of the genome (Figure 6B and Figure S2). TE depletion in these sensitive genomic areas is much more pronounced for protein-coding genes than for lncRNA genes. This is in agreement with the overall greater contribution of TEs to lncRNA exons (Figure 2), but it suggests that the proximal flanking regions of lncRNA loci are also enriched in TEs relative to protein-coding genes. This trend is most apparent for LTR/ERV elements in human, which are strongly depleted in the vicinity of protein-coding genes but in relatively high abundance for intergenic lncRNAs in the exons and proximal regions of lncRNAs (Figure 6B and 6D and Figure S2A and S2B). Consistent with this relative enrichment of LTR/ERV elements, we found that nearly all of the statistically most enriched TE families in human lncRNA exons and upstream regions belong to the LTR/ERV class (both ERV internal regions and their LTRs, Figure S3A and S3B and Figure 7A). Moreover, 42.5 and 45% of TE-derived TSS in the Gencode v13 and Cabili lncRNA sets respectively map within ERVs (Table S4). Together these data indicate that LTR/ERV make a greater contribution to human lncRNAs and their upstream flanking regions than other types of TEs.

Interestingly, we also found a relative enrichment of a majority of LTR/ERV elements in exons and proximal regions of mouse Ensembl lincRNAs (Figure S3C and Figure 7). This is similar to human, even though their lncRNAs are largely non-orthologous [9], [10] and their associated LTR/ERV elements mostly belong to lineage-specific families (Figure S3). These data therefore point to a convergent process whereby LTR/ERV elements are enriched in exons and upstream regions of human and mouse lncRNA genes.

### TEs, and LTRs in particular, contribute many likely cis-regulatory elements controlling lncRNA transcription

Given the relative abundance of TEs in the first exon and upstream regions of lncRNA genes, we sought to better evaluate the contribution of TEs to the cis-regulation of lncRNA transcription. To do this, we repeated the analysis described above with a subset of human TEs inferred to have cis-regulatory potential based on their positional overlap with DNaseI hypersensitive sites (DHS) clusters mapped as part of the ENCODE project [81], [82] (see Methods). Such DHS clusters are reliable indicators of active chromatin and are enriched for regulatory proteins such as transcription factors [81], [83]. We identified a total of 35,263 TEs with putative cis-regulatory activity, hereafter designated as DHS-TEs, within or in the vicinity (10 kb upstream or downstream) of Gencode v13 lncRNA loci. Consistent with cis-regulatory function, we found that DHS-TEs are significantly enriched in the 1-kb window upstream of lncRNA and protein-coding genes (compare Figure 6A and 6B). DHS-TEs are also enriched in lncRNA exons (Figure 6B), suggesting that these elements are likely involved in cis-regulation of lncRNA transcription.

For protein-coding genes, the greatest enrichment of DHS-TEs is observed for SINEs located in the proximal (<1 kb) upstream region (Figure 6B). However for lncRNAs, the greatest enrichment of DHS-TEs involve LTRs located in the proximal upstream region, where their density is about twice as high compared to the rest of the genome (Figure 6B). Together these data point to the widespread implication of TEs, and in particular LTRs, to the cis-regulation of human lncRNA genes.

**Figure 8:**
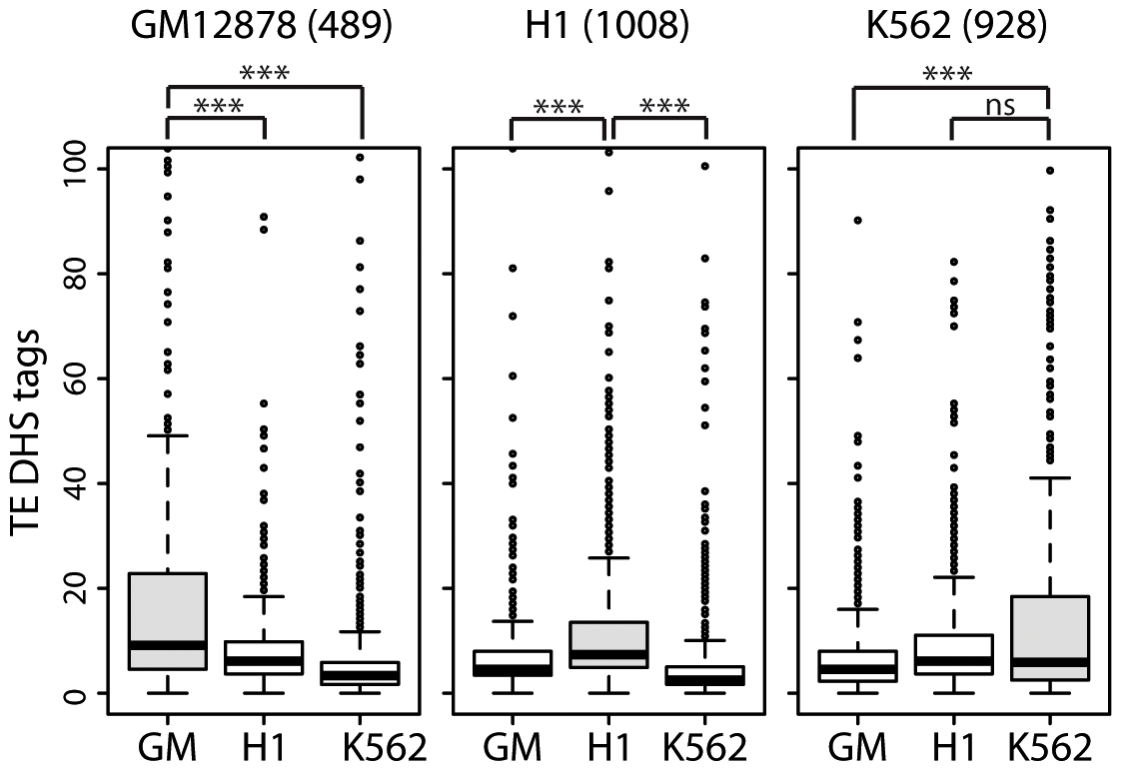
lncRNAs with cell-type specific expression are also associated with cell-type specific TE–DHS. Cell-type specific lncRNA based on RNA-Seq expression (cutoff of 10-fold higher) were identified in GM = GM12878, H1 or K562. Numbers of cell-type specific lncRNAs are written above graphs. For each lncRNA, only the most active proximal TE-DHS (<10 Kb) was retained and the distribution of normalized tag counts over these elements are shown in each cell type (*** for *P*<0.0001, ns for *P*>0.5.).

To further assess the cis-regulatory activity of TEs upstream of lncRNAs, we assembled subsets of lncRNAs with cell-type specific expression in one of three human cell lines (489 lncRNAs in GM12878, 1008 in H1 and 928 in K562) for which RNA-seq data was generated as part of the ENCODE project (see Methods). We examined the level of activity of TE-DHS in the upstream region (<10 kb) of these cell-type specific lncRNAs and looked for evidence of cell-type specific regulation. Notably we found that lncRNAs that are highly expressed in a given cell type are also associated with more active TE-DHS mapped in the same cell type (Figure 8). These results indicate that the opening of chromatin in a TE located in the upstream region of a lncRNA locus correlates with high level of lncRNA transcription in a cell type-specific fashion. Together these analyses suggest that TEs located in the vicinity of hundreds of lncRNA loci carry the hallmarks of cis-regulatory elements and some appear to provide cell type-specific enhancer elements controlling adjacent lncRNA expression.

### TEs promote the lineage-specific diversification of lncRNAs

Because transposition represents a major source of lineage-specific DNA, we wanted to evaluate its contribution to the evolution of the vertebrate lncRNA repertoire. Our examination of TE-derived sequences in studied human lncRNAs reveals that many of these elements are restricted to primates (36.3% for Gencode v13, Figure S4), suggesting that TEs play an important role in the diversification and possibly the birth of primate-specific lncRNAs. Few of the human lncRNAs functionally characterized have identifiable orthologs in non-primate species, but *Xist* and *cyrano* provide solid examples of functional lncRNAs with ancient evolutionary origins. *Xist* is involved in X-chromosome inactivation and originated in the common ancestor of eutherian mammals [45], [46]. Previous *in silico* reconstruction of the *Xist* locus in the eutherian ancestor suggested that several TEs were already present at the dawn of the *Xist* gene and likely contributed to the assembly of the first functional *Xist* transcript [46]. Other TEs embedded in *Xist* exons are lineage-specific and therefore must have contributed to the diversification of the transcript during eutherian evolution. For example, a primate-specific FLAM_C element makes up nearly half (114 nt) of the first *Xist* exon in human (Table S2).

**Figure 9:**
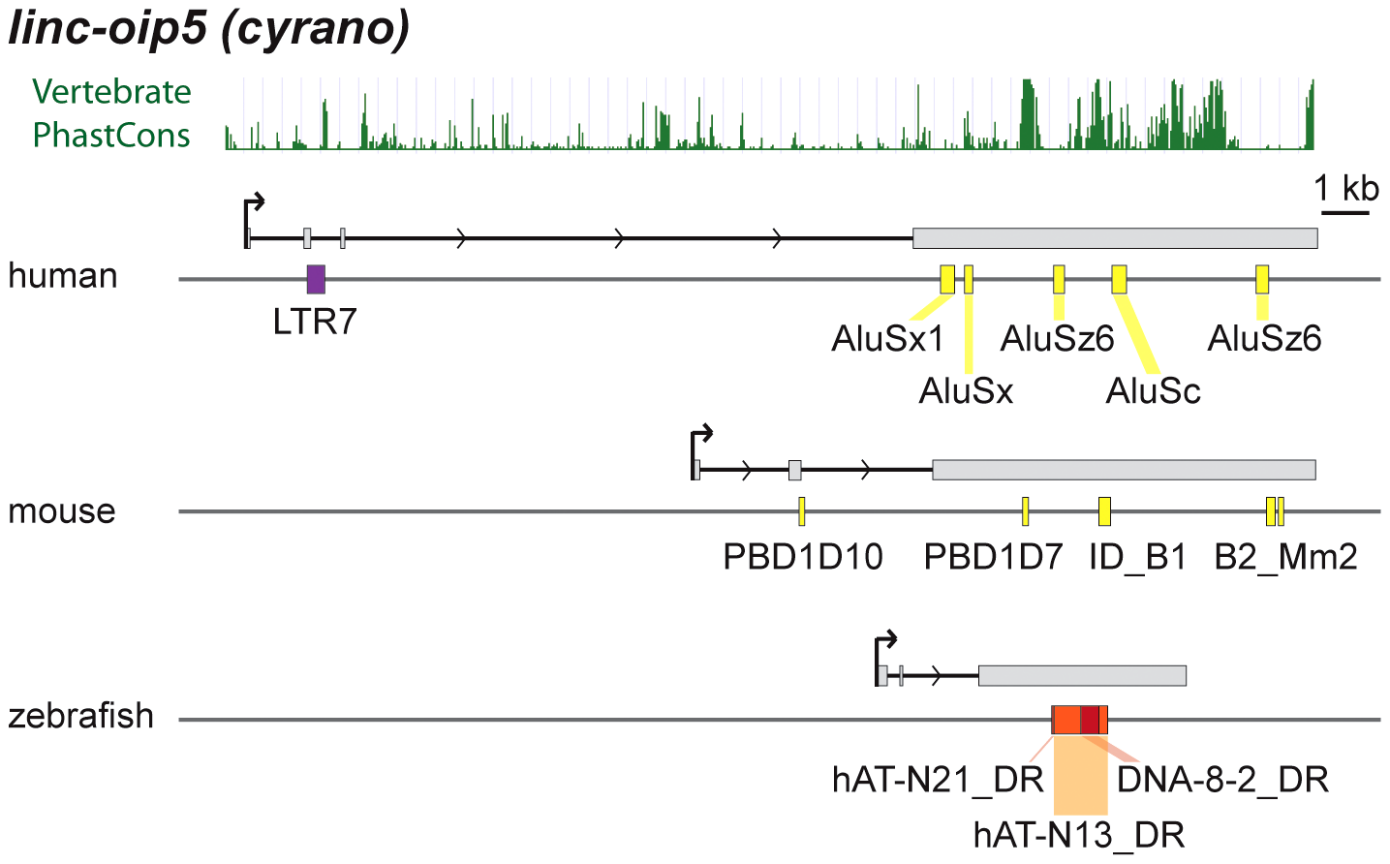
Lineage-specific TE insertions in *cyrano*. Symbols and graphics are as in Figure 3. The structure of *cyrano* (*lnc-oip5*) [14] is based on coordinates of Gencode v13 transcript *OIP5-AS1-001*. Vertebrate PhastCons: peaks of sequence conservation across 46 vertebrate genomes displayed in the UCSC genome browser.

*cyrano* is one of a small subset of zebrafish lncRNAs sharing significant sequence similarity and synteny with apparent orthologs in mouse and human [14]. Most of the sequence similarity between species is limited to a central region of the last exon (see PhastCons plot in Figure 9). In zebrafish embryos, *cyrano* is expressed in the nervous system and notochord and morpholino-mediated knockdowns followed by rescue experiments indicate that this lncRNA plays a role in neurodevelopment, a function possibly conserved in mammals [14]. We find that the conserved exonic region of *cyrano* is flanked by lineage-specific TEs embedded in this orthologous exon in each of the three species examined (Table 2, Figure 9). These examples illustrate how TEs can be incorporated long after the birth of lncRNAs to diversify their sequence in a lineage-specific fashion.

Among functionally characterized human lncRNAs, we uncovered numerous instances where the TSS resides in primate-specific TEs (Table S2). In most of those cases, the TE provides the only identified TSS for that lncRNA locus, suggesting a pivotal role for these TEs in the biogenesis and most likely the birth of these lncRNAs during primate evolution. These include six of the eight known lncRNAs containing the largest TE amounts listed in Table 2, which all have their TSS located within the LTR of an ERV element. Intriguingly, these instances include two different lncRNAs that are highly expressed in human embryonic stem cells (ESCs) and have been experimentally shown to be implicated in the maintenance of ESC pluripotency: *lncRNA-RoR* [41] and *lncRNA-ES3* [43]. The transcripts cloned for *lncRNA-RoR* and *lncRNA-ES3* both initiate within LTR7/HERVH elements (Figure 3B and 3C). Furthermore, we found that these same LTR7 elements have donated the DNA binding sites for the ‘master’ transcriptional regulators of pluripotency NANOG, OCT4, and SOX2 mapped previously to the proximal promoter of *lncRNA-RoR* [41] (Figure 3B). Ng et al. [39] mapped two binding sites for NANOG in the promoter region of *lncRNA-ES3* that we find to reside within the LTR7 driving this locus (Figure 3C). The contribution of LTR7 to the regulation of these lncRNAs in ESCs is consistent with two recent studies showing that TEs, and LTR/ERV elements in particular, play an extensive role in the primate-specific wiring of the core transcriptional network of human ESCs [57], [84]. In fact, Kunarso et al. [57] identified LTR7/HERVH as one of the most over-represented TE families seeding OCT4 and NANOG binding sites throughout the human genome. Our results indicate that this ERV family also contributed to the recruitment of primate-specific lncRNAs into the pluripotency network of human ESCs [see also ref. 7].

### Some TEs confer lncRNAs the potential to form secondary structures

**Figure 10:**
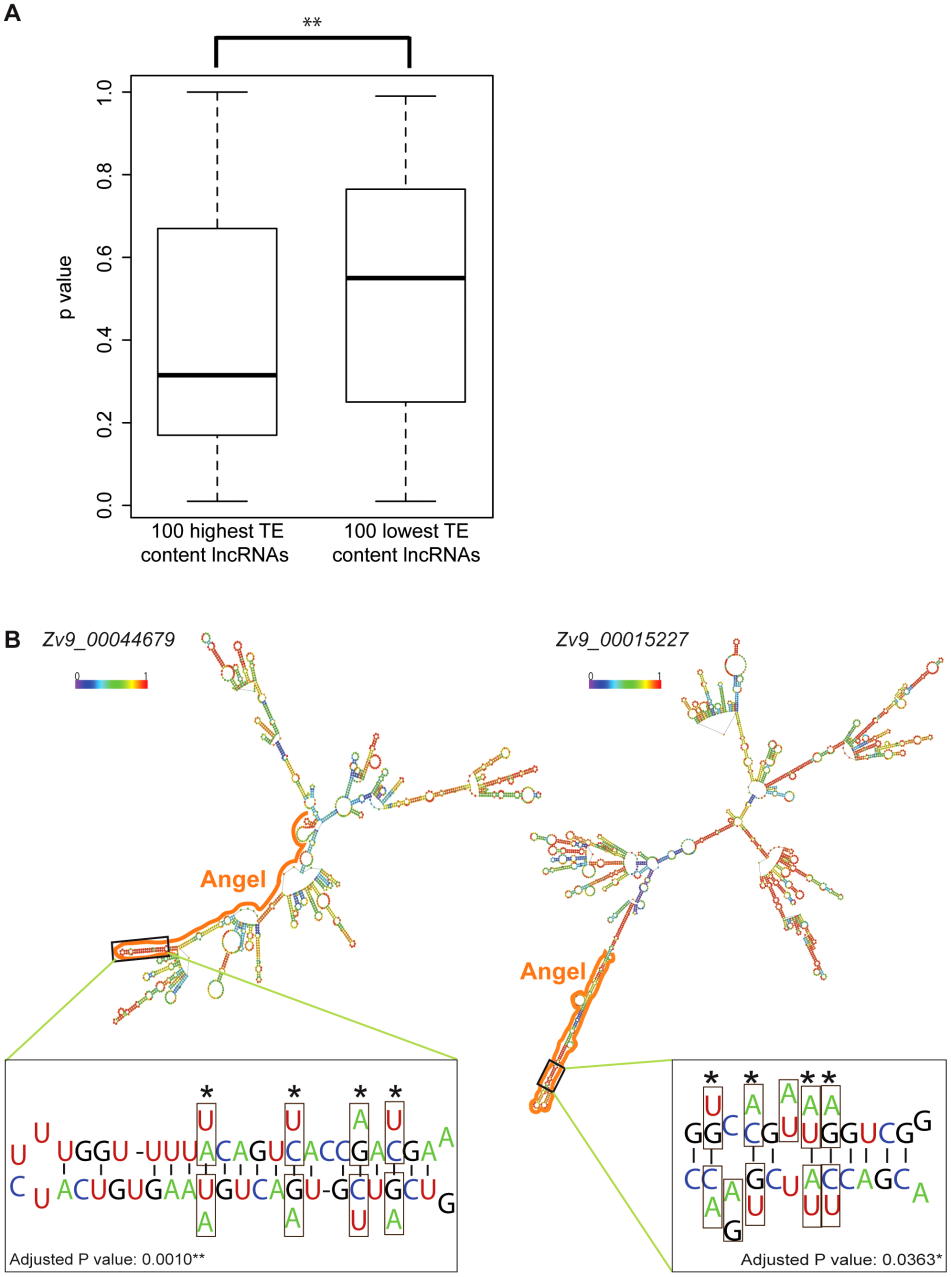
TE contribution to predicted lncRNA secondary structures. A. High and low TE content groups of 100 lncRNA were extracted from Gencode v13 set (TE content from 96.74% to 100% and from 0.49% to 2.27% respectively; see Table S7). P-values were calculated by Randfold and provide an indication of predicted secondary structure stability. The boxplot depicts the maximum, upper quantile, median, lower quantile and minimum value in a standard way. The mean of these 2 groups are significantly different by Wilcox rank sum test (p = 0.0022). B. Predicted secondary structures (RNAfold [115]) and compensatory mutations for two zebrafish lncRNAs containing ANGEL (DNA TEs) elements. In structures, TE derived regions are marked by solid line and base pairing probability by color spectrum (from 0 in violet to 1 in red). Zoom-in windows show part of stem with compensatory mutations: nucleotide substitution are boxed and the corresponding nucleotide found in ANGEL consensus are shown under/above actual RNA sequence. Sites of compensatory mutations are marked by asterisks and written p-values are adjusted by Bonferroni methods.

Since lncRNAs act at the RNA level, we hypothesized that TEs may participate in the folding of lncRNAs into secondary structures, which could be important for their function. One prediction of this hypothesis is that lncRNAs with high TE content may fold into more stable structure than those with low TE content. To test this, we selected from the Gencode v13 set the top 100 lncRNAs with highest TE content and the top 100 lncRNAs with lowest TE content (see Methods) and compared the minimum free energy (MFE) of predicted secondary structures computed by the program *randfold* [85] for each of these individual lncRNAs. For each input sequence, *randfold* attributes a *p*-value to a predicted MFE by comparing it with a MFE obtained for the same sequence randomly reshuffled 99 times (See ref. [85] and Methods). The average *p*-value for high TE content lncRNAs was significantly lower than the one of low TE content lncRNAs (p = 0.0022, Wilcox rank sum test) (Figure 10A). The average length of the lncRNAs in the two datasets was also substantially different (913 nt and 1,913 nt for high and low TE content respectively), but there was no correlation between RNA length and *p*-value for the 200 lncRNAs examined (data not shown), ruling out a possible bias introduced by lncRNA length. Together these results indicate that TEs generally stabilize lncRNA structure in human, which supports the hypothesis that some of the TEs embedded in lncRNA exons contribute to the folding of lncRNAs into secondary structures.

To explore further this hypothesis, we studied a family of DNA transposons in zebrafish, called *Angel*, which occur in high copy numbers and are known to have the potential to form a stable stem-loop structure at the RNA level due to their long inverted repeats [86]. We reasoned that the incorporation of *Angel* elements in lncRNAs might in some case have conferred a functional benefit by increasing RNA stability. We identified 71 zebrafish lncRNAs containing exonized *Angel* elements. As expected, RNA folding programs predict that these lncRNAs have the potential to form a long stem-loop structure by intramolecular pairing of the *Angel* inverted repeats (see examples in Figure 10B). Furthermore, by comparing the sequence of these elements to that of their ancestral (consensus) progenitor, we identified two instances of *Angel* elements in lncRNAs where base substitutions in one of the arms of the predicted stem-loop structure were accompanied by compensatory substitutions on the other arm allowing the maintenance of base-pairing within the stem-loop structure (Figure 10B). To rule out the possibility that these substitutions occurred not at these loci, but prior to transposon insertion in a progenitor element that would have amplified or duplicated, we used BLAT [87] to search the zebrafish Zv9 genome assembly for paralogous *Angel* elements that might be sharing the same substitutions. In each case, we found that the compensatory substitutions we identified were unique to the *Angel* copies residing within the examined lncRNAs (data not shown), suggesting that these mutations occurred after transposon insertion. The probability that these compensatory substitutions would have occurred by chance alone in these two *Angel* elements is 0.001 and 0.036 after Bonferroni correction, respectively (see Methods). Furthermore, 12 of the 16 concerted mutations were from A/T to C/G base pairs, which is consistent with the idea that they increased the stability of the stem-loop structure. These data suggest that these *Angel* elements indeed fold into the predicted secondary structures *in vivo* and have been maintained over time by natural selection, plausibly for the proper function of the lncRNAs.

To seek another, independent line of evidence for the involvement of TEs in forming secondary structures potentially important for lncRNA function, we next looked for sites of adenosine-to-inosine (A-to-I) editing in lncRNAs. This form of RNA editing is catalyzed by the ADAR family of adenosine deaminases that act on double-stranded RNA templates [88]. In humans, it has been reported that A-to-I RNA editing occurs predominantly within *Alu* elements embedded in the 3′ UTR of protein-coding transcripts [89]–[91]. This bias has been explained by the relatively high frequency of *Alu* elements in transcribed regions of the human genome, which often occur in inverted pairs and thereby can form long RNA duplexes providing templates for ADARs [92].

**Figure 11:**
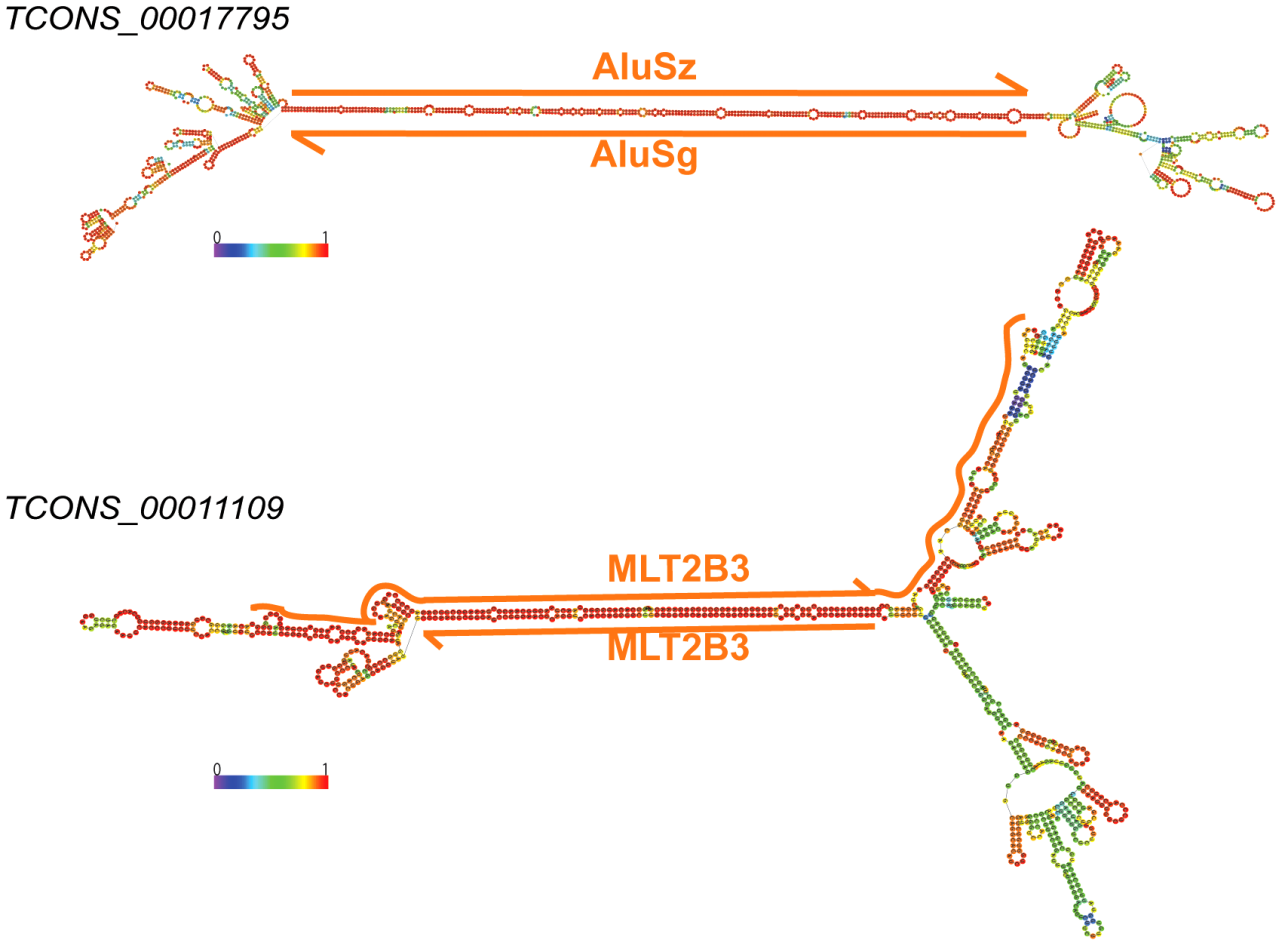
Long stem in two human lincRNAs (Cabili set) formed by inverted TEs. Two examples of heavily edited human lincRNA transcripts with editing sites located in TEs. RNA structures are predicted by RNAfold [115]. Nucleotide color in structures indicates base pairing probability (from 0 to 1). Inverted TE pairs are marked by solid lines, the arrow illustrating TE strand. The stem pair of *TCONS_00017795* is composed by inverted Alu elements, while the structure of *TCONS_0001109* is formed by 2 LTRs (MLT2B3).

We used DARNED, a database of RNA editing sites in humans [93], to identify 2,941 A-to-I editing sites in mature lncRNA transcripts. As observed previously for mRNAs, most (82%) of the edited sites in lncRNAs occur within *Alu* elements, although we also found evidence of A-to-I editing within a wide range of TE types embedded in lncRNAs (Table 3 and Table S6). This may be explained by the fact that non-*Alu* TE sequences are much more frequent in lncRNAs than in mRNAs, even when UTRs are considered separately (Figure 2 and Figure S1). Indeed, we found that the density of edited sites within *Alu*, non-*Alu* TE, or non-TE sequences fall within the same order of magnitude in lncRNAs and UTRs (Table 3). In several cases individually examined, we found that editing sites in TE sequences map preferentially within regions of the lncRNA predicted to form stem-loop structures by virtue of the inclusion of two inverted copies of the same TE family in the lncRNA (see two examples in Figure 11). The finding that TE sequences, and in particular *Alu* elements, embedded in lncRNAs are frequent templates for A-to-I editing confirms that TEs are commonly engaged in intra- or inter-molecular base pairing interactions to form stable dsRNA structures.

**Table 3:** Editing sites in exons.

Total editing sites	lincRNA	5′UTR	3′UTR
Alu	1521	29	5297
other TE	162	0	121
nonTE	187	87	688

Editing sites per 100 kbp#	lincRNA	5′UTR	3′UTR
Alu	298	59	472
other TE	6.19	0	6.84
nonTE	3.23	1.75	3.17

# Editing sites density in Alu and non-Alu sequences are similar among non-coding transcript. The density of Alu in different sequences are about 10-3, while in other TE elements and non TE sequences the density is about 10-5.

## Discussion

Recent high-throughput efforts to characterize the transcriptome of multicellular eukaryotes have uncovered thousands of lncRNA genes [5]–[7], [17]–[19]. While current lncRNA catalogs, such as those we used, are still far from exhaustive and almost certainly contain false positives, they indicate that the abundance and complexity of lncRNAs in mammalian genomes may rival or exceed that of protein-coding genes [9], [10], [13], [16]. The precise functions of the vast majority of lncRNAs remain to be determined, but evidence from genetic, genomic, and biochemical experiments, as well as analyses of sequence constraint, suggest that many lncRNAs perform important functions, most notably in the control of protein-coding gene expression during development and differentiation [for review: 5], [6], [7]. Despite the functional importance of some known lncRNAs, the basic mechanisms of lncRNA evolution have been largely unexplored. The few studies that have examined the evolutionary dynamics of lncRNAs paint a picture of evolutionary volatility, where large cohorts of lncRNAs seem to appear, disappear, or rapidly diversify, pointing to a potentially important role of lncRNAs in lineage-specific regulatory innovation [6], [10], [16]. Here we present a systematic assessment of TE contribution to the makeup, evolutionary origins, and regulation of vertebrate lncRNAs. While this paper was in its final stage of preparation, a study by Kelley and Rinn [94] analyzed in some detail the contribution of TEs to human and mouse multi-exonic intergenic lncRNAs. The two studies complement each other in that we analyzed different lncRNA sets, but arrive at the same general conclusion that TEs are important players in the composition and diversification of lncRNAs, highlighting a new way mobile elements have influenced genome evolution and shaped a likely crucial layer of genomic regulation.

### TEs are ubiquitous in vertebrate lncRNAs, but the type and amount of TE vary among species

While TEs are seldom found in protein-coding transcripts (even in their UTRs, see Figure 1 and Figure 2), they are ubiquitous in lncRNAs of all three vertebrates examined (Figure 1A), accounting for a large fraction of total lncRNA sequence (Figure 2). Thus, high TE prevalence is probably a common characteristic of vertebrate lncRNA repertoires that distinguish them from mRNAs and smaller ncRNAs, such as tRNAs or microRNAs, which are typically TE-depleted (Figure 1A).

We found that all major TE classes are found in lncRNAs in each of the three vertebrate species surveyed, and their relative abundance mirrors that of the entire genome (Figure 2). Nonetheless, in each species we identified TE families that were statistically enriched (up to 32 fold) in lncRNA exons relative to their coverage or density in the whole genome (Figure 7 and Figure S3). Interestingly, these over-represented families belong to different TE classes in the species examined, for example, LTR/ERV in human and mouse and DNA transposons in zebrafish (compare colors per species in Figure 7 and Figure S3). The predominance of DNA transposons in zebrafish is expected based on the prevalence of DNA transposons in this genome (see Figure 2, Figure 7 and Figure S1). However our results show that LTR/ERVs contribute disproportionally to lncRNAs in human and mouse, which is in agreement with the recent results reported by Kelley and Rinn [94].

Interestingly, human lncRNAs are mostly enriched for the ERV I subclass (alpharetroviruses), compared to mouse where ERV 2, ERV 3 or ERV K TEs are enriched (Figure 7 and Figure S3). ERV 1 subclass of elements is less abundant in the mouse genome [95] and strongly repressed in mouse ESCs [96], [97]. Therefore, it is not surprising that this type of retroviral elements do not contribute more to mouse lincRNAs. While LTR/ERV elements are also generally silenced in most human tissues, a subset of families is known to escape silencing and to become transcriptionally active in some tissues, cell types, or at certain developmental stages [54], [98]–[100]. These properties may derive from the intrinsic capacity of retroviruses to hijack host transcriptional activators in order to promote their own expression in a cell-type or developmentally restricted fashion [51], [52], [55]. For example, hundreds of ERV I elements recruit the pluripotency factors OCT4 and NANOG in human ESCs, but rarely do so in mouse ESCs [57]. This mechanism can readily explain why *lncRNA-RoR* and *lncRNA-ES3* and hundreds of other lncRNAs associated with ERV I elements (such as *LTR7/HERVH*) are highly transcribed in human ESCs (Figure 3, Table 2) [see also ref. 94]. This trend is also globally apparent through the enrichment of LTR elements (including LTR7/HERVH) in 286 human lncRNAs upregulated in ES cells [annotations from Table S1, 10] (data not shown). In sum, the interspecific variations we observe in the coverage and type of TEs in lncRNAs likely reflect a variety of factors; both methodological, such as the breadth of cell types and tissues examined, and biological such as the abundance and intrinsic properties of certain TEs residing in the genome.

### “lncRNA first” versus “TE first”: Divergence or emergence?

Two scenarios can explain the prevalence of TEs in lncRNAs. The first is that TE insertion in pre-existing lncRNAs has relatively little deleterious effect on lncRNA function allowing TEs to accumulate over time as waves of transposition break in the genome. We call this scenario the ‘lncRNA first’ model because it implies that the origin of the lncRNA predates the incorporation of TE(s) in their exons. In the second and not mutually exclusive scenario, the ‘TE first’ model, lncRNAs are assembled from TEs that inserted before the birth of the lncRNAs. Several observations and examples outlined below indicate that both models contribute to the pervasive occurence of TEs in lncRNAs.

The “lncRNA first” scenario is supported by a comparison of the few lncRNAs known to be of relatively ancient origin, exemplified by *Xist* or *cyrano*, which have assimilated lineage-specific TE insertions sequentially during evolution (see Figure 9 and Table S2). Typically, these exonized TEs correspond to the most variable regions of the transcript sequence flanking more deeply conserved core sequences (see [14] and Figure 9). On a broader scale, we observe that TEs predominantly contribute to the last exon of lncRNAs (56.5% of TE amount, see Figure S5). The biased incorporation of TEs to the 3′ region of transcripts is also apparent for mRNAs, where exonized TEs are more abundant in 3′ UTRs (Figure 1, Figure 2 and Figure S5), as reported previously [65]–[67]. These data suggest that TE-derived sequences are preferentially acquired at the 3′ end of pre-existing transcripts, either because this region is more permissive to TE insertion and/or because TEs are somehow predisposed for this type of exonization events, for example owing to the presence of cryptic acceptor splice sites facilitating their capture [70], [101], [102]. In any case, this 3′ bias is consistent with the ‘lncRNA first’ model whereby TEs are secondarily acquired by existing lncRNAs.

On the other hand, several observations support the ‘TE first’ model. First, we identified thousands of lncRNA transcripts that are mostly or entirely composed of TEs (Figure 1B). It is difficult to conceive that these lncRNAs would have emerged from ancestral non-TE regions later replaced or obliterated by secondary TE insertions. More likely, these lncRNAs were born from material providing by pre-existing TE insertions. In support to this idea, we identified 4,404 human Gencode v13 lncRNA transcripts with TE-derived TSS, with 1,777 of these (40.4%) derived from primate-specific TE families (Table S4). In addition, we found 2,213 human lncRNA transcripts whose first exons are entirely derived from TEs, and 965 of these (43.6%) are derived from primate-specific TE families (Table S4). These values are very similar when only genes with a unique TSS are considered and we retrieved comparable numbers in the Cabili set (Table S4). Since these TEs provide the only TSS assigned for these transcripts, we propose that these lncRNAs were born from the transcriptional activity brought upon TE insertion in the genome. Interestingly, 36.8% (857/2,331) of the TE-derived unique TSS map within LTR/ERV elements, while this type of elements account for only 8% of all TEs in the human genome (see Figure 2). Thus, it appears that the tissue-specific transcriptional activity of LTR/ERV elements [52], [54], [100] represents a major force driving the birth of lncRNAs. These data also imply that a substantial fraction of human lncRNAs are of recent origin, because ∼40% of TEs driving human lncRNAs are primate-specific and some are even restricted to hominoids (e.g. Figure 3A and 3B, Table S2).

In summary, our data suggest that, in some instances, TE insertion events have been a source of diversification of ancestral lncRNAs, while in others TE insertions have triggered the emergence of brand new lncRNAs during evolution. In order to better quantify the relative importance of either process to lncRNA evolution, it will be necessary to infer systematically the age of lncRNAs using a comparative RNA-seq approach [16].

### TE–mediated regulation of lncRNA genes

It has been extensively documented that mammalian TEs represent an abundant source of cis-regulatory sequences driving or modulating the expression of adjacent protein-coding genes [reviewed in 49], [56]. Our study provides evidence that TEs located in the vicinity of lncRNAs may also frequently contribute to the transcriptional regulation of these genes. As discussed above, LTR/ERV elements appear to make a disproportionate contribution to lncRNA regulation relative to other TE types and in some cases they may be solely responsible for the cell-type specificity of lncRNA expression. This is exemplified by *lncRNA-RoR* whose transcription in hESCs is driven by a LTR7/HERVH element occupied by the pluripotency factors OCT4, NANOG and SOX2 (Figure 3C and [41], [94]). Thus, much like LTR/ERV elements have been implicated in the wiring of protein-coding genes into specific regulatory networks [55], [57], [59], [84], they have also recruited lncRNAs serving important developmental function, notably in the pluripotency network [41], [43], [94].

### Possible functions of TEs embedded in lncRNAs

Perhaps the most pressing question to address in the future is to what extent TEs may contribute to the function of lncRNA and how? Our analysis shows that TEs embedded in lncRNAs frequently supply sequences and signals essential for the transcription (e.g. TSS) and processing (e.g. splice, polyA sites) of the lncRNAs (Figure 5). However it does not prove that TE sequences *per se* are indispensable for lncRNA function, if such function even exists. Many studies have used various approaches and statistics to show that lncRNA exons, as a whole, display weak but significant signals of purifying selection suggesting that at least a fraction of lncRNA sequences is subject to functional constraint detectable at the primary DNA level [10], [12], [29]–[33]. Our analysis confirms the existence of a signal of purifying selection acting on human lncRNA exons, but more importantly we observe that this signal is higher in TE-derived than in non-TE derived lncRNA sequences (Figure 4A) suggesting that a subset of TE sequences in lncRNAs are structurally or functionally constrained.

TE-derived sequences could serve as the functional elements of lncRNAs in numerous ways. For example, TE sequences might provide interaction interfaces with proteins involved in post-transcriptional or transcriptional regulation, such as the chromatin modifiers often found in complex with lncRNAs [37], [103]. Their inclusion may also provide opportunities for base-pairing interaction with single-stranded DNA or RNA containing similar repeats in inverted orientation. Such duplexes might act as a platform to recruit protein effector complexes to genomic or RNA targets. For example, *Alu* elements embedded within several human lncRNAs form a group called 1/2sbs-RNAs that base-pair with complementary *Alu* elements located in the 3′-UTR of several protein-coding transcripts to form duplexes creating a binding site for the Staufen1-mediated RNA decay machinery, which in turn promote post-transcriptional repression of the targeted mRNAs [104]. Given the abundance of *Alu* and other high copy number TEs in lncRNAs, such trans-regulatory effects may be widespread and affecting a large number of mRNAs containing complementary TEs in their UTRs. It was also shown recently that a B2 SINE embedded in a mouse lncRNA antisense to *Uchl1* is required for post-transcriptional up-regulation of UCHL1 protein synthesis, an activity that can be transferred to an artificial antisense green fluorescent protein transcript containing the B2 SINE element [105]. We identified 361 mouse lncRNAs containing B2 SINEs (16.7%; see Tables S5 and S9), raising the possibility that these elements confer similar post-transcriptional regulatory activity to other lncRNAs.

Finally, another recent study identified a point mutation associated with a lethal form of infantile encephalopathy within a primate-specific LINE-1 retrotransposon transcribed as part of a lncRNA in the human brain [78]. The precise function of the LINE-1 element in this lncRNA is unknown, but knockdown of the lncRNA resulted in increased neuronal apoptosis, an effect consistent with the encephalopathy phenotype. Interestingly, the point mutation detected in affected individuals was predicted to destabilize a secondary structure in the corresponding lncRNA, suggesting that the LINE-1 element may contribute to lncRNA folding that is important for its function in the brain. Similarly, we identified several instances in zebrafish and in human where TEs embedded in lncRNAs are predicted to be involved in the formation of stem-loop structures that have been maintained in evolution through compensatory mutations and therefore are likely to be functionally significant. We also found that these structures often lead to RNA editing of lncRNAs, which to our knowledge is a novel observation that may be relevant to the function of some lncRNAs [88], [92]. We also show that human lncRNAs fold into more stable structure than those with low TE content, suggesting that these individual examples of TEs apparently co-opted for the cellular function of lncRNAs likely represent only the tip of a large iceberg. Future work is bound to unravel a variety of mechanisms through which TEs embedded in lncRNAs have become involved in regulating the expression of vertebrate genomes.

### Conclusions

There is growing evidence that vertebrate genomes contain a large number of long non-coding RNA genes (lncRNAs) that play important gene regulation roles, however, remarkably little is known about the origins of these genes. Our study reveals that TEs, through their capacity to move and spread in genomes in a lineage-specific fashion, as well as their ability to introduce regulatory sequences upon chromosomal insertion, represent a considerable force shaping the lncRNA repertoire of human, mouse and zebrafish. These results suggest that the apparent paucity of ancient lncRNA genes may be explained in part by rapid turnover mediated by lineage-specific TEs and imply that the regulatory networks in which lncRNA genes act may be rapidly diverging between species.

## Methods

### lncRNA datasets

The datasets used in this study are as follow: human, Gencode release 13 (from ftp://ftp.sanger.ac.uk/pub/gencode) and Cabili et al. (2011) [9]. Mouse, Ensembl release 70 (ftp://ftp.ensembl.org/pub/release-70/gtf/mus_musculus/) and Kutter et al (2012) [16], both sets filtered to keep only intergenic lncRNAs. Coordinates from Kutter et al. were converted from mm9 to mm10 using the liftover tool from UCSC (http://genome.ucsc.edu/cgi-bin/hgLiftOver?hgsid=325693955). Zebrafish sets are from Pauli et al. (2012) [24] and Ulitsky et al. (2011) [14]. To limit redundancy, in case of overlap of exons between transcripts of the two sets, only transcripts from Pauli et al. (2012) were kept. Additional descriptors of the datasets are provided in Table 1.

### TE annotation

TE annotations used in this study are obtained from the outputs of the RepeatMasker (RM) software [106] produced for the following genome assemblies: human, hg19 assembly, RM v.330, repbase libraries 20120124, from RM website (http://www.repeatmasker.org/species/homSap.html). Mouse, mm10 assembly, RM v.330, repbase libraries 20110920, from UCSC website (http://hgdownload.soe.ucsc.edu/goldenPath/mm10/bigZips/). Zebrafish, danRer7: RM v.329, repbase libraries RB20090604, from UCSC website (http://hgdownload.cse.ucsc.edu/goldenPath/danRer7/bigZips/). These RM outputs were filtered to remove non-TE elements (Low Complexity, Satellites, Simple Repeats and ncRNA). For mouse, MutSatRep1, CYRA11_Mm and YREP_Mm are also removed. To minimize multiple counting of single TE copies artificially fragmented in the RepeatMasker outputs we merged consecutive pieces of the same TE separated by less than 10 nt.

### Counts of TEs in transcripts

The TE content of lncRNA transcripts (datasets described above) and human RefSeq 57 ncRNAs (22,486 in total), pseudogenes (13,430), CDS and UTRs (20,848 protein coding genes) was determined by intersecting these sets with each species' TE annotations (described above) using the ‘Table Browser’ at the UCSC Genome Bioinformatics Site (http://genome.ucsc.edu/index.html) [107]. Only overlaps of minimum 10 bp were kept.

### Coverage of TEs in exons and surrounding sequences

Protein-coding gene (pc genes) models were filtered to retain only those with 5′ and 3′ UTRs, from following releases: human, Refseq 49 (hg19, gtf file from UCSC genome browser); mouse, Refseq 57 (mm10, gtf file from UCSC genome browser); zebrafish, Ensembl 68 (danRer7). All nucleotide amounts correspond to genomic amount. Introns and upstream or downstream intergenic regions were processed through Galaxy [108]–[110] to remove all RefSeq genes exons (CDS and UTRS: Refseq 51 for human and zebrafish, Refseq 57 for mouse) as well as lncRNA exons of the datasets considered. Intergenic sequences (upstream or downstream, up to 10 or 1 kb) correspond to the longest fragment between TSS or polyA and another feature (RefSeq entries as well as lncRNA exons of the dataset considered). These sets (exons, introns, intergenic sequences) were then joined in Galaxy with filtered RepeatMasker outputs described above keeping only fragments with at least 10 nt of overlap, to calculate TE coverage of exons. See Tables S7, S8, S9, S10, S11 for transcripts TE content data.

### Conservation PhyloP

By comparing their PhyloP scores across an alignment of 10 primate genomes, the conservation of human (Gencode v13) TE-derived lncRNA exonic segments was compared to non-TE derived lncRNA segments, RefSeq 57 5′- and 3′-UTRs, protein-coding exons and a set of random genomic fragments size-matched to the TE-derived lncRNA segments. We also generated a set of TE-derived lncRNA intronic segments, non overlapping with splicing sites and corresponding to inactive chromatin to obtain a most neutral set to compare with exonic TE-derived lncRNA segments [32] (all annotated chromatin marks from 9 cell lines were subtracted: GM12878, H1-hESC, HMEC, HSMM, HUVEC, HepG2, K562, NHEK, NHLF; ENCODE version Jan. 2011). Precompiled PhyloP scores were obtained from the ‘phyloP46wayPrimates’, ‘phyloP46wayPlacental’, and ‘phyloP46wayAll’ tracks available from the UCSC Genome Bioinformatics Site (http://genome.ucsc.edu/index.html) [107] and intersected with gene annotations using bedtools (http://code.google.com/p/bedtools/) [111]. Boxplots were made in R (http://www.r-project.org). Statistical test used: permutation test with 1000 permutations were performed in R.

### TE contribution to functional features: Exon counts

TEs were not assigned a strand allowing them to overlap genomic features on either strand. TEs found in a genomic feature were classified based on their position in the feature, as schematized in Figure 5A. Both lncRNA and protein-coding genes were filtered before both the random and non-random analyses. In case of multiple splice forms a random mRNA was kept. Additionally protein coding genes that did not have both a 5′ UTR and 3′ UTR were excluded from the analysis. Sets of protein-coding genes are as follows: human, Refseq release 49, mouse, Refseq release 46, zebrafish, Ensembl release 68. For the random sets, all TEs were shuffled within chromosomes (excluding gaps) while preventing TE overlap. This process was repeated 5,000 times for each set, using a custom perl script (see http://www.yandell-lab.org/publications/index.html). The standard error for the random sets across all categories (Figure 5A) always plateaued before 1,000 replicates (data not shown). The probability of observing the non-random counts was calculated using the random sets. The p-value represents number of times a lower category count was observed in the random set out of 5,000 replicates. With the exception of ‘exonized’, ‘TSS’ and ‘polyA’ categories in mouse (p-values = 1, 0.001 and 0.298 respectively) and ‘exonized’ category in zebrafish (p-value = 0.001), the p-values were systematically <0.0001.

### Spatial correlation between TEs and genes

The GenometriCorr (Genometric Correlation) package from R [112] was used to test the degree of overlap between TEs and genomic features (protein coding exons, lincRNAs) [Exploring Massive, Genome Scale Datasets with the GenometriCorr Package]. This package uses the Jaccard distance. The Jaccard distance measures the overlap between two sets of genomic ranges (A & B) compared to the total genomic range A and B occupy. Jaccard distance ({A},{B}) = |{A}∩{B}|/|{A}U{B}|. To test if the observed overlap is statically significant, one set of genomic features (TEs) were shuffled 1,000 times and the Jaccard distance was taken for each permutation.

### Overlap with DNaseI clusters and cell-type-specific regulation/TEs set

We made use of the DNase I clusters track from the integrated ENCODE data sets [81], [82], [113], which was retrieved from the UCSC Genome Browser [107]. The Dnase clusters were intersected with our list of annotated TEs using the program BEDTools [111] and TEs overlapping by more than 10 bp a Dnase cluster were retained. We also retrieved paired 2×75 bp RNA-Seq data sets from ENCODE/Caltech in GM12878, H1 and K562 cell lines. Expression of lncRNAs was measured using BEDTools by calculating the coverage over the length of the lncRNAs and was normalized by the total number of reads in each cell line. We identified cell-type specific lncRNAs as those having a 10-fold higher expression in a given cell type relative to the average expression in the other two cell-types. Next, to look at cell-type specific regulation we made use of the University of Washington DNase I ENCODE data sets from the same cell lines. Total coverage of reads was calculated over the length of TEs in proximity to lncRNAs (<10 Kb) using the program BEDTools to measure accessibility in each cell-type and was normalized by the total number of reads in each library. For each lncRNA, only the most active TE in each cell line was retained for analysis. P-values for the significance of the differences between the distributions were calculated using a Wilcoxon rank sum test.

### Randfold

The top 100 lncRNA transcripts with highest TE content (from 100% to 96.74%) and the top 100 lncRNAs with lowest TE content (from 2.27% to 0.49%) in the Gencode v13 set (see Table S7) were used as input for randfold [85]. They were reshuffled 99 times with dinucleotide shuffling option. Wilcox rank sum test was used to test whether the average p-value of high TE content lncRNAs is smaller than the value of low TE content lncRNAs.

### Compensatory mutations

All lncRNAs with ANGEL elements coverage in exon region were extracted for compensatory mutation identification. A Perl script was used to compare each ANGEL in lncRNA to the ANGEL consensus in Repbase [114]. lncRNAs with putative compensatory mutations were manually examined and RNA structures predicted using RNAfold [115]. With a transition/transversion ratio of κ, when 2 mutations occur on a same base pair the probability of 2 mutations being compensatory is:
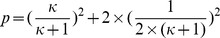
κ: the transition/transeversion ratio in zebrafish. We are using κ = 1.2 based on SNP analysis in zebrafish [116]. And assume mutations occur on a short stem follows hypergeometric distribution, the probability of having as much as observed compensatory mutation in the pairing stem is:
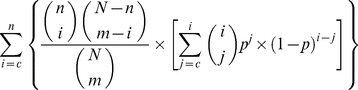
c: compensatory mutations observed;

m: mutations observed on a strand in a pairing stem;

n: mutations observed on another strand in a pairing stem;

N: total pairing nucleotides in the pairing stem.

p: probability of compensatory mutation if 2 mutations occur in a pair of bases.

Significance was calculated using R language (http://www.r-project.org) and the p-value was adjusted by the Bonferroni correction.

### RNA editing

We intersected genomic coordinates of lincRNAs from Cabili set and protein-coding transcripts (5′UTR, coding region and 3′ UTR analyzed separately) coordinates with human RM output as described above. This allowed annotation of segments as “non-TE”, “Alu-derived” and “non-Alu TE-derived”. that we intersected with editing sites compiled in the DARNED database [93]. Heavily edited lincRNAs were extracted and their secondary structures predicted with RNAfold [115].

## Supporting Information

Figure S1 Coverage of different TE classes in genome, lncRNA and protein-coding exons of human, mouse and zebrafish. Values are the same as in Figure 2, but 100% corresponds here to total amount of TEs.
(TIF)

Figure S2 TE amounts and counts in lncRNA surrounding regions, by class of TE. Counts correspond to the percentage of a given TE class, 100% being total number of TEs overlapping with a given dataset (see Methods). Coverage is calculated as described for Figure 6 and in Methods. Counts and coverage are shown per TE class (LTR, nonLTR/LINE, nonLTR/SINE, DNA) with an additional separation between LTRs (LTR/LTR) and internal parts (LTR/int) of LTR elements. Regions are: genome, intergenic regions and exons. In the case of protein coding genes, exons include UTR exons as well as coding exons. 1 or 10 kb up and dw = intergenic regions up to 1 or 10 kb upstream of the TSS and downstream of the polyA respectively. Any annotated exons (RefSeq and lncRNA sets) have been subtracted from intergenic and intronic regions. A. Human, Gencode v13, TE counts only, for all TEs and DHS TEs (coverage is in Figure 6). B. Human, lincRNAs from Cabili et al (2011). C. Mouse, lincRNAs from Ensembl release 70 and Kutter et al. (2012) [16]. D. Zebrafish. DNA TEs are also split into 3 different classes (hAT, TcMar and Others).
(TIF)

Figure S3 Over represented TE families in lncRNAs. The expected and observed amounts of DNA corresponding to each TE are calculated using Repeat Masker output (see Methods). Observed values are obtained by considering overlapping TEs lncRNA exons or promoter regions. Expected values are calculated based on the null hypothesis that different TE families in lncRNA exons undergo the same selection pressure. The significance of enrichment (counts) is calculated based on binomial distribution (* for *P*<0.05, ** for *P*<0.01, *** for *P*<0.001). Only statistically over represented TE families (test on counts) are kept. For human sets, TEs with less than 5 fragments in lncRNAs are removed, 4 fragments for mouse and zebrafish. Either all families with a ratio >1 or the 25 most over represented are shown. A. Human, set from Gencode v13. B. Human, set of Cabili et al. (lincRNAs). C. Mouse, lincRNAs from Ensembl. D. Zebrafish.
(TIF)

Figure S4 Amount of lineage specific and ancient TEs in human lncRNAs and protein-coding genes genomic environment. G: genome. See Methods “Coverage of TEs in exons and surrounding sequences” for details on sets. Ancient TEs correspond to TEs shared between placental mammals (Eutherians). Gencode v13 set for lncRNA.
(TIF)

Figure S5 Relative amount of TEs depending on exon type. TE contribution means that 100% is the total coverage of a given class of TEs. For example, ∼20% (19.5) of TE amount is in first exon for lncRNAs, whereas for pc genes it is 6.4% (“All” TEs). lncRNAs are Gencode v13 set. A. Coverage. B. Counts.
(TIF)

Table S1 TE coverage and TE counts in genic and intergenic human lncRNAs and surrounding regions.
(XLSX)

Table S2 Detailed TE content of known lncRNAs presented in Table 2.
(XLSX)

Table S3 Numbers of exons overlapping with TEs (data corresponding to Figure 5).
(XLSX)

Table S4 Counts of functional features provided by ancient or primate TEs in human.
(XLSX)

Table S5 Editing sites in Cabili lincRNA set.
(XLSX)

Table S6 361 mouse lincRNAs with B2 SINEs elements (ENSEMBL release70).
(XLSX)

Table S7 Details of TE content of human Gencode v13 lncRNAs.
(XLSX)

Table S8 Details of TE content of human lincRNAs from Cabili et al. (2011).
(XLSX)

Table S9 Details of TE content of mouse lincRNAs from ENSEMBL release 70.
(XLSX)

Table S10 Details of TE content of mouse lincRNAs from Kutter et al. (2012).
(XLSX)

Table S11 Details of TE content of zebrafish lincRNAs.
(XLSX)
