# Supplementary figures and images for: Transposable Elements Are Major Contributors to the Origin, Diversification, and Regulation of Vertebrate Long Noncoding RNAs
Source: PLoS Genet. 2013 Apr 25;9(4):e1003470. doi: 10.1371/journal.pgen.1003470 (PMC3636048; doi:10.1371/journal.pgen.1003470)

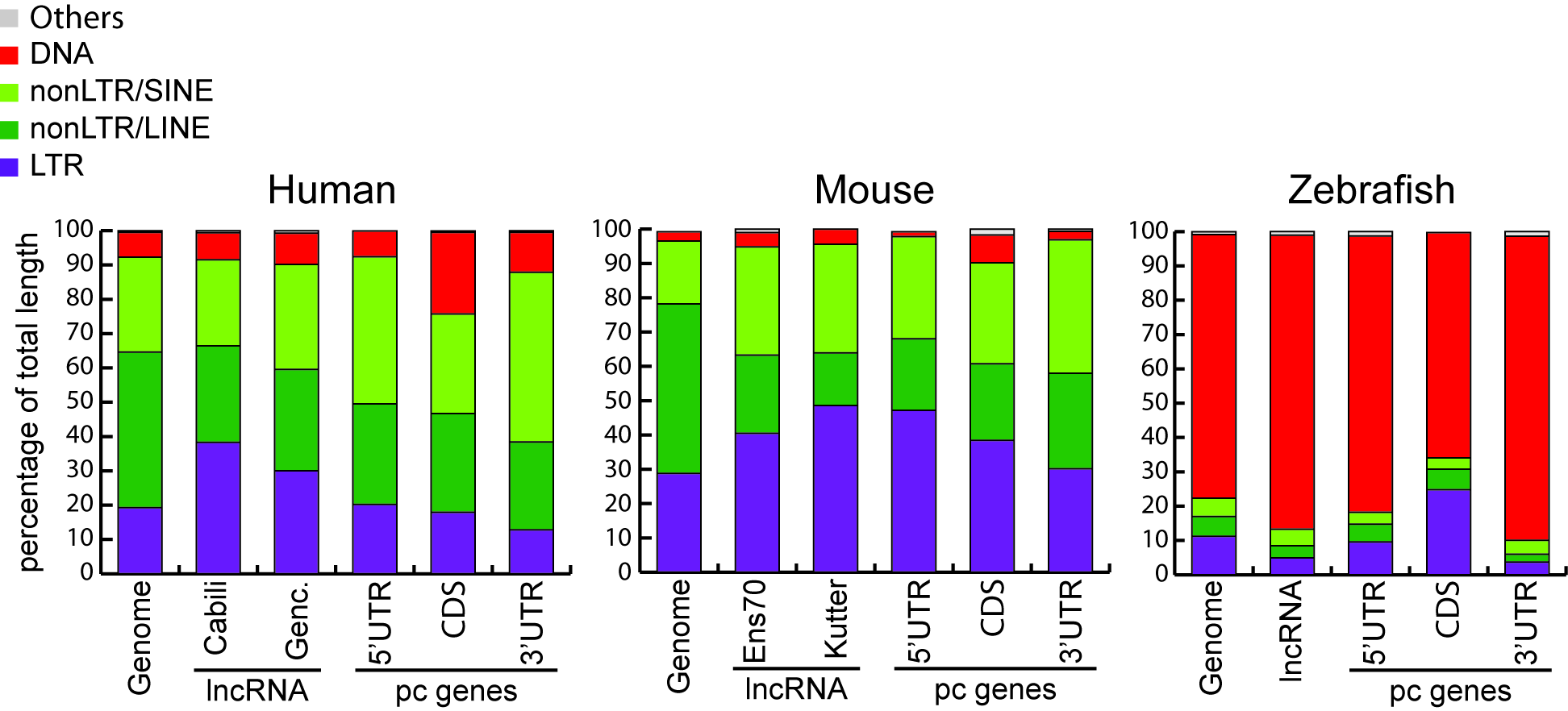

Supplement: Figure S1 — Coverage of different TE classes in genome, lncRNA and protein-coding exons of human, mouse and zebrafish. Values are the same as in Figure 2, but 100% corresponds here to total amount of TEs. (TIF) [file pgen.1003470.s001.tif]

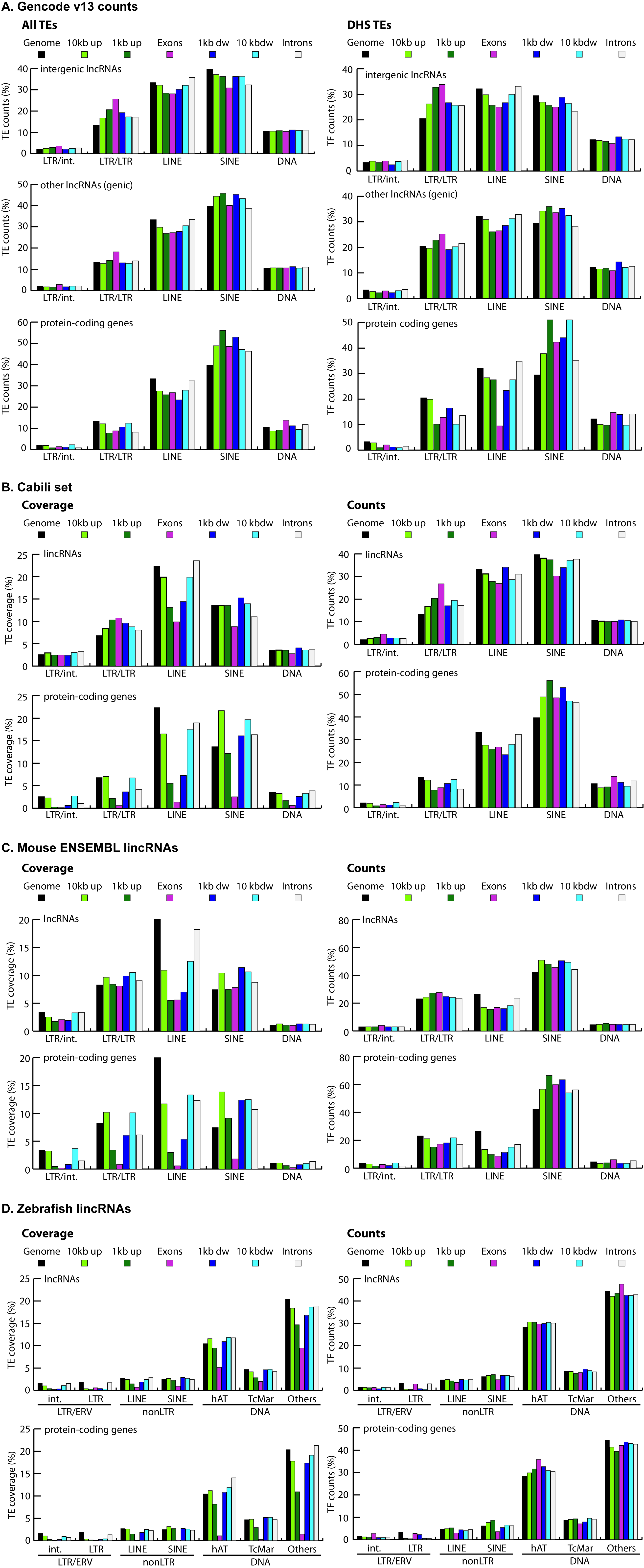

Supplement: Figure S2 — TE amounts and counts in lncRNA surrounding regions, by class of TE. Counts correspond to the percentage of a given TE class, 100% being total number of TEs overlapping with a given dataset (see Methods). Coverage is calculated as described for Figure 6 and in Methods. Counts and coverage are shown per TE class (LTR, nonLTR/LINE, nonLTR/SINE, DNA) with an additional separation between LTRs (LTR/LTR) and internal parts (LTR/int) of LTR elements. Regions are: genome, intergenic regions and exons. In the case of protein coding genes, exons include UTR exons as well as coding exons. 1 or 10 kb up and dw = intergenic regions up to 1 or 10 kb upstream of the TSS and downstream of the polyA respectively. Any annotated exons (RefSeq and lncRNA sets) have been subtracted from intergenic and intronic regions. A. Human, Gencode v13, TE counts only, for all TEs and DHS TEs (coverage is in Figure 6). B. Human, lincRNAs from Cabili et al (2011). C. Mouse, lincRNAs from Ensembl release 70 and Kutter et al. (2012) [16]. D. Zebrafish. DNA TEs are also split into 3 different classes (hAT, TcMar and Others). (TIF) [file pgen.1003470.s002.tif]

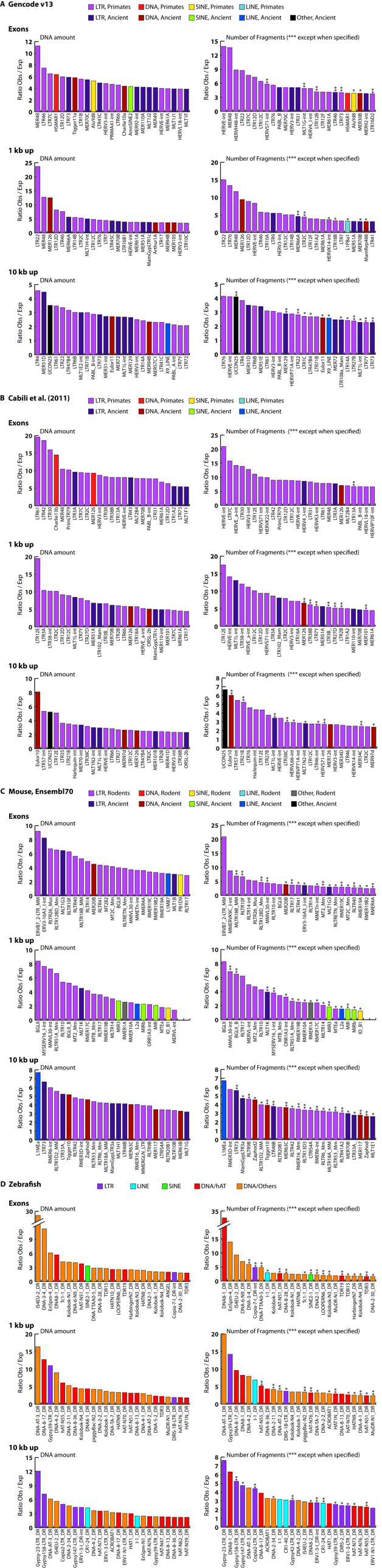

Supplement: Figure S3 — Over represented TE families in lncRNAs. The expected and observed amounts of DNA corresponding to each TE are calculated using Repeat Masker output (see Methods). Observed values are obtained by considering overlapping TEs lncRNA exons or promoter regions. Expected values are calculated based on the null hypothesis that different TE families in lncRNA exons undergo the same selection pressure. The significance of enrichment (counts) is calculated based on binomial distribution (* for P<0.05, ** for P<0.01, *** for P<0.001). Only statistically over represented TE families (test on counts) are kept. For human sets, TEs with less than 5 fragments in lncRNAs are removed, 4 fragments for mouse and zebrafish. Either all families with a ratio >1 or the 25 most over represented are shown. A. Human, set from Gencode v13. B. Human, set of Cabili et al. (lincRNAs). C. Mouse, lincRNAs from Ensembl. D. Zebrafish. (TIF) [file pgen.1003470.s003.tif]

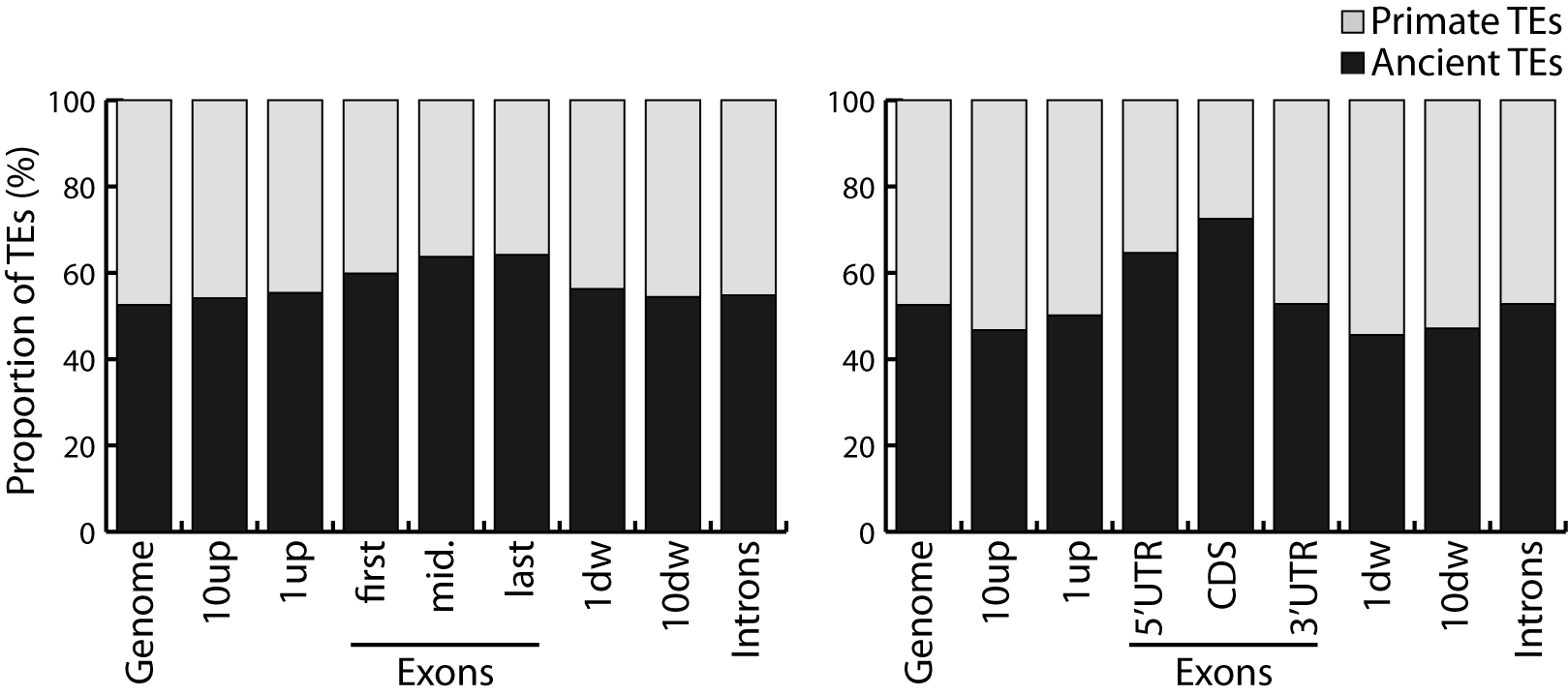

Supplement: Figure S4 — Amount of lineage specific and ancient TEs in human lncRNAs and protein-coding genes genomic environment. G: genome. See Methods “Coverage of TEs in exons and surrounding sequences” for details on sets. Ancient TEs correspond to TEs shared between placental mammals (Eutherians). Gencode v13 set for lncRNA. (TIF) [file pgen.1003470.s004.tif]

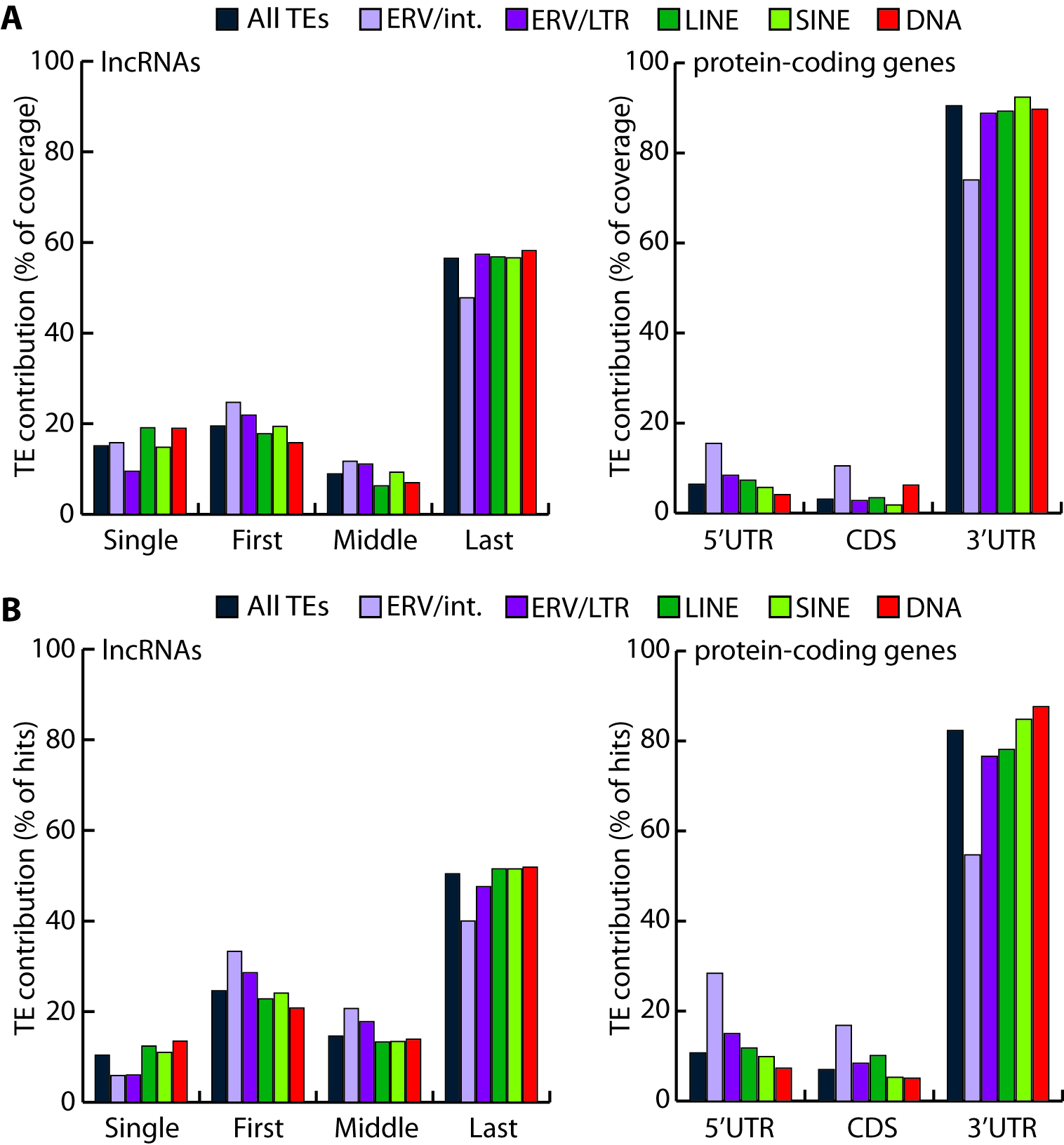

Supplement: Figure S5 — Relative amount of TEs depending on exon type. TE contribution means that 100% is the total coverage of a given class of TEs. For example, ∼20% (19.5) of TE amount is in first exon for lncRNAs, whereas for pc genes it is 6.4% (“All” TEs). lncRNAs are Gencode v13 set. A. Coverage. B. Counts. (TIF) [file pgen.1003470.s005.tif]
